# TNS1/TAGLN-enriched endometrial stromal cells is associated with a persistent fibrotic microenvironment in intrauterine adhesion

**DOI:** 10.3389/fcell.2026.1810255

**Published:** 2026-08-13

**Authors:** Li Tang, Yutang Li, Siyao Chen, Xueyu Zhao, Shengnan Jiang, Bingzi Jia, Yingli Lu

**Affiliations:** 1 Department of Obstetrics and Gynecology, The Second Hospital of Jilin University, Changchun, Jilin, China; 2 Department of Burn Surgery, The First Hospital of Jilin University, Changchun, Jilin, China

**Keywords:** fibrosis, human endometrial stromal cell, intrauterine adhesion, oxidative stress, tensin 1, transgelin

## Abstract

Intrauterine adhesion arise from aberrant repair and fibrotic remodeling after basal endometrial injury. Hysteroscopic adhesiolysis remains the standard treatment, yet postoperative recurrence stays high and durable preventive strategies remain limited. Most studies analyze IUA at the tissue level or in mixed cell populations, leaving the key pathological states and regulatory nodes of endometrial stromal cells insufficiently defined. Here, we integrated bulk transcriptomic data (GSE165321) and single-cell transcriptomic data (GSE215968) from IUA patients and validated findings using a TGF-β1–induced *in vitro* fibrotic model of human endometrial stromal cells and an *in vivo* rat IUA model. Differential expression and co-expression network analyses, together with protein–protein interaction mapping and functional annotation, identified TNS1 and TAGLN as key IUA-associated genes. Single-cell analyses showed marked upregulation of TNS1 and TAGLN in stromal cells. Virtual knockout suggested that TNS1 perturbation mainly affected adhesion/calcium-dependent and ion-transport programs, whereas TAGLN perturbation triggered broader alterations in metabolic and inflammation-related pathways; TNS1 knockout also coincided with potential compensatory upregulation of TAGLN. *In vitro*, TGF-β1 induced a fibrotic phenotype (increased COL1A1, FN1, ACTA2 and reduced COL3A1; elevated fibronectin and α-SMA) and increased TNS1 and TAGLN at both mRNA and protein levels. Flow cytometry showed a higher proportion of TNS1^+^TAGLN^+^ cells, accompanied by enhanced collagen-gel contraction and elevated intracellular ROS and mitochondrial superoxide. In the rat model, endometrial destruction, gland loss, and collagen deposition progressed over time. The result of immunohistochemistry, immunofluorescence, and Western blot revealed stage-dependent stromal expression: TNS1 changed most prominently at 4–6 weeks, whereas TAGLN continued to increase at 6–12 weeks. Collectively, our study suggests a pathological stromal cell state characterized by high TNS1/TAGLN expression in IUA, accompanied by adhesion–contraction programs, metabolic/inflammatory perturbations, and oxidative stress, which may help sustain a pro-fibrotic microenvironment and promote disease progression.

## Introduction

Intrauterine adhesion (IUA), also known as Asherman syndrome (AS) (Asherman, 1948), represent a fibrotic or myogenic adhesive disorder of the uterine cavity that arises from injury to the basal layer of the endometrium followed by aberrant repair processes. IUA frequently develops after mechanically induced endometrial trauma, such as submucosal myomectomy, induced abortion, or dilation and curettage (Wu et al., 2024). In addition to mechanical injury, intrauterine infection and inflammation serve as important contributing factors to disease onset (Hooker et al., 2021). This condition leads to a broad spectrum of reproductive complications, including infertility, recurrent pregnancy loss, dysmenorrhea, hypomenorrhea, and secondary amenorrhea (Lee et al., 2021; Salazar et al., 2017). Compared with healthy women, patients with IUA also face significantly increased risks of early miscarriage, preterm birth, and fetal growth restriction, thereby imposing a substantial burden on female reproductive health (Carbonnel et al., 2021).

At present, hysteroscopic adhesiolysis remains the standard treatment for IUA (Evans-Hoeker and Young, 2014). However, recent meta-analyses indicate that postoperative recurrence rates exceed 50% (Munro et al., 2025). To reduce recurrence risk, clinicians have widely implemented various adjuvant strategies in recent years, including estrogen supplementation (Cao et al., 2020), intrauterine devices, bioactive hydrogels (Wang et al., 2021), stem cell–based therapies (Chen et al., 2023), and exosome-based approaches (Wang et al., 2023). Despite these advances, no available intervention effectively lowers the recurrence rate of IUA. Consequently, elucidating the cellular and molecular mechanisms that govern IUA development and recurrence remains a pressing clinical need.

Under normal physiological conditions, HESCs regenerate the functional layer of the endometrium under hormonal regulation, thereby maintaining tissue integrity and reproductive capacity. This regenerative process depends on coordinated epithelial–stromal interactions and tightly controlled inflammatory responses (Gargett et al., 2016; Feng et al., 2024). Once damage disrupts the basal layer of the endometrium, aberrant wound repair programs become activated. These processes involve persistent inflammation, induction of epithelial–mesenchymal transition (EMT) (Thiery et al., 2009), activation of fibrosis-related signaling pathways such as TGF-β/Smad (Salma et al., 2016), excessive extracellular matrix (ECM) deposition (Zhao et al., 2017), and disruption of immune homeostasis (Yao et al., 2023). As a result, luminal epithelial cells are largely lost, stromal cell regenerative capacity declines, normal endometrial tissue becomes replaced by fibrotic tissue, and IUA ultimately form (Owusu-Akyaw et al., 2019). Although previous studies have extensively described these pathological events, most investigations rely on tissue-level analyses or bulk cell populations, leaving the specific cell types and state transitions that actively drive fibrotic progression insufficiently characterized.

The broad application of single-cell RNA sequencing (scRNA-seq) has enabled high-resolution analysis of cellular heterogeneity during tissue repair and fibrotic remodeling. HESCs differentiate from endometrial mesenchymal stem/stromal cells (HESCs) with stem-like properties and function as the principal architects of endometrial connective tissue. These cells synthesize and secrete type I and type III collagens, providing mechanical support and structural stability for endometrial glands, vasculature, and surrounding cellular compartments (Tanaka et al., 2008; Matsuzaki et al., 2023). Previous studies demonstrate that HESCs isolated from patients with IUA exhibit reduced migratory, invasive, angiogenic, and immunomodulatory capacities compared with those from healthy women (Min et al., 2021). However, current research primarily emphasizes functional impairment of HESCs, while whether these cells undergo lineage or state shifts during endometrial injury and repair remains poorly explored. To date, it remains unclear whether specific stromal cell states closely associate with excessive ECM deposition and sustained pro-fibrotic microenvironments.

Therefore, by integrating bulk RNA sequencing data and single-cell transcriptomic datasets from patients with IUA, together with *in vitro* and *in vivo* functional experiments, we identified a previously unrecognized pathological endometrial stromal cell state that shows marked enrichment in IUA. This stromal cell state features elevated expression of *Tensin* 1 (TNS1) and *Transgelin* (TAGLN), responds to TGF-β signaling, and participates in cytoskeletal remodeling, mechanotransduction, and oxidative stress responses. Through these coordinated properties, this cell population provides critical environmental support that exacerbates endometrial fibrosis and promotes IUA recurrence. These findings offer new mechanistic insight into endometrial fibrogenesis and establish a theoretical foundation for the development of precision-targeted therapeutic strategies.

## Methods

### Overall study design and analytical workflow

**FIGURE 1:**
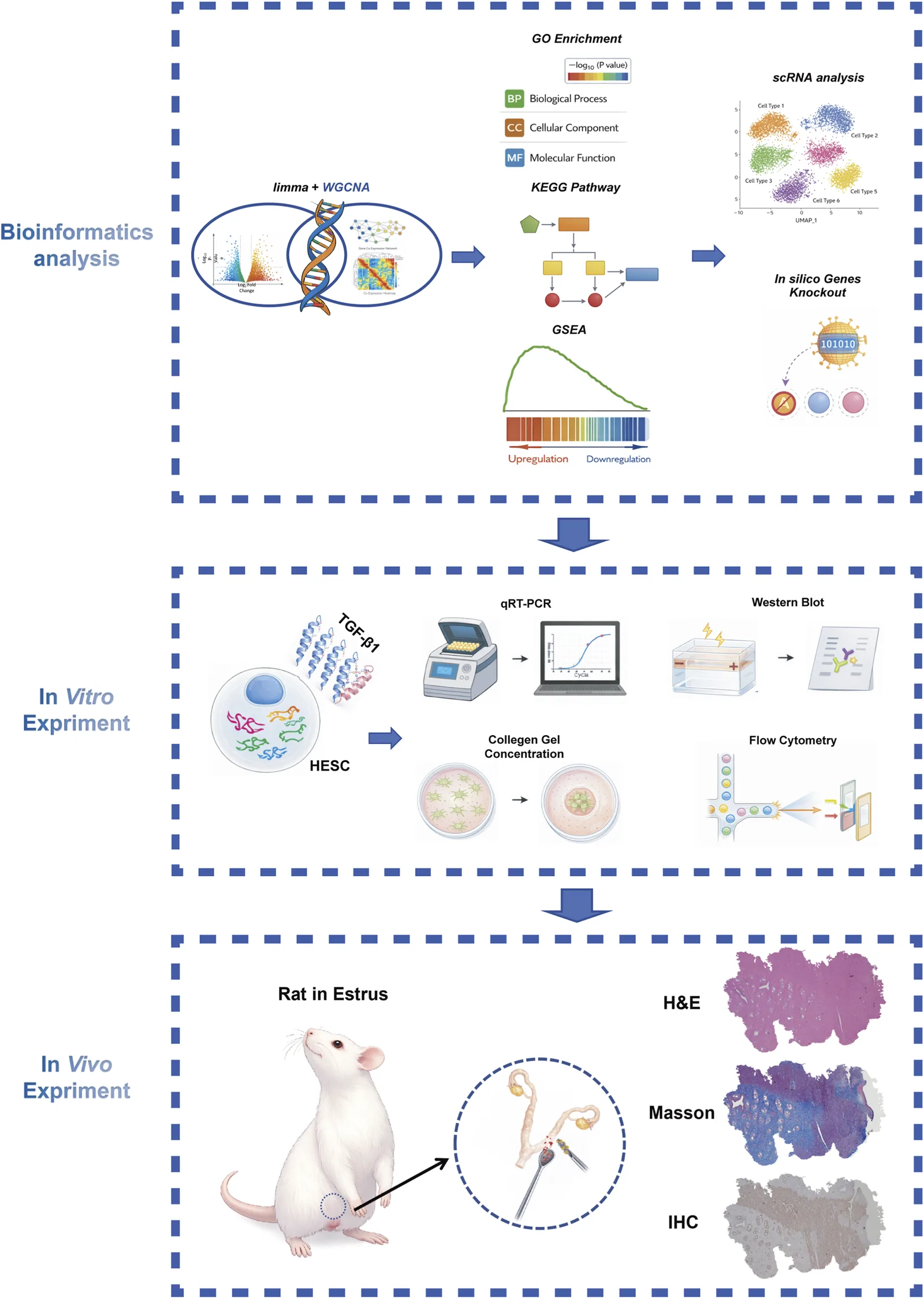
The research process is illustrated in the Figure 1 and is divided into 3 parts: Bioinformatics analysis, *in vitro* experiments, and *in vivo* experiments.

This study first integrated bulk RNA sequencing data (GSE165321) and single-cell RNA sequencing data (GSE215968) from patients with IUA. Differential expression analysis, co-expression network analysis, protein–protein interaction mapping, and pathway enrichment analyses were performed to identify key regulatory genes associated with IUA, followed by single-cell–level characterization of endometrial stromal cell heterogeneity and virtual knockout analyses to assess the potential functional consequences of TNS1 and TAGLN perturbation. Next, functional validation was conducted using a TGF-β1–induced *in vitro* fibrosis model of HESCs by assessing fibrotic markers, expression of TNS1 and TAGLN, the proportion of TNS1^+^TAGLN^+^ cells, collagen gel contraction, and intracellular oxidative stress and mitochondrial functional parameters. Finally, a rat IUA model was established to evaluate endometrial structural alterations, gland loss, and collagen deposition across different disease stages. Western blot, immunohistochemical quantification and immunofluorescence quantification were performed to examine the temporal expression patterns of TNS1 and TAGLN in the endometrial stromal compartment (Figure 1).

### Data collection and key genes identification

The transcriptomic datasets GSE165321 and GSE215968 were retrieved from the Gene Expression Omnibus (GEO) database (https://www.ncbi.nlm.nih.gov/geo). As summarized in Table 1.

**TABLE 1:** The information of GEO series.

Matrix	Country	Disease	Sample	Experimental group	Control group
GSE165321	China	IUA	Total RNA of endometrium	3 IUA	3 health
GSE215968	Spain	IUA	Single-cell RNA sequencing	17 IUA (untreated), 6 health control	

Differentially expressed genes (DEGs) were identified using the limma package in R (version 4.3.2) (Ritchie et al., 2015). Genes with an absolute log fold change (|logFC|) ≥ 1 and an adjusted *p* < 0.05 were considered significantly differentially expressed and visualized using volcano plots. To further identify gene modules associated with IUA, Weighted Gene Co-expression Network Analysis (WGCNA) was performed (Langfelder and Horvath, 2008). Genes with excessive missing values and those in the lowest 25% of expression levels were removed prior to network construction. The soft-thresholding power was selected based on the scale-free topology criterion (*R* ^2^ > 0.85) across candidate powers ranging from 1 to 20. Modules with highly correlated eigengenes (correlation ≥0.85) were merged, and modules containing fewer than 300 genes were excluded.

Module–trait relationships were evaluated by calculating gene significance (GS) and module membership (MM) scores. Modules with a correlation coefficient >0.5 and *p* < 0.05 were defined as key modules. Genes with |GS| ≥ 0.9 and |MM| ≥ 0.9 (*p* < 0.05) were considered hub genes associated with IUA. A significant correlation between GS and MM within each key module was required to confirm biological relevance.

Overlapping genes identified by limma and WGCNA were visualized using a Venn diagram. Protein–protein interaction (PPI) networks were constructed using the STRING database with a minimum interaction score of 0.3. Functional annotations of intersecting genes were obtained from the GeneCards and OMIM databases.

### Functional enrichment analysis

To explore the biological functions of IUA-associated genes, Gene Ontology (GO) and Kyoto Encyclopedia of Genes and Genomes (KEGG) enrichment analyses were performed using the Bioinformatics and Omics Data Analysis platform. Enrichment terms with *p* < 0.05 were considered statistically significant, and the results were visualized using bubble plots [https://www.bioinformatics.com.cn/].

Gene Set Enrichment Analysis (GSEA) was conducted using the clusterProfiler package in R (version 4.3.2). The top six enriched pathways, ranked by normalized enrichment score (NES), were visualized using enrichment plots (*p* < 0.05).

### Single-cell RNA-seq verification and analysis

The single-cell RNA sequencing dataset GSE215968 was processed using the Seurat package (version 4.4.1) in R. Quality control filtering was performed to retain cells with nFeature_RNA >500 and mitochondrial gene percentage (percent.mt) < 5%. Cell clustering was conducted with the FindClusters function at a resolution of 0.5, and the resulting clusters were visualized with Uniform Manifold Approximation and Projection (UMAP) for dimensionality reduction. Cell type annotation was performed using the SingleR package, referencing the Human Primary Cell Atlas dataset provided in the celldex package. To characterize the cellular distribution of key genes, the expression patterns of the identified hub genes across major endometrial cell types in healthy controls and IUA patients were visualized using a dot plot. Furthermore, the differential expression of these hub genes specifically within HESCs was illustrated using violin plots. To investigate the impact of target gene perturbation, we performed virtual knockout (KO) experiments using the R package scTenifoldKnk (Osorio et al., 2022), a machine learning workflow based on scRNA-seq data. This approach allowed us to identify genes perturbed by the virtual KO of target genes. Differentially significant genes (*p* < 0.05) were subjected to functional enrichment analysis.

### HESC culture and fibrotic model establishment

The experimental cells were immortalized human endometrial stromal cells (IHESC-SV40), purchased from Zhejiang Meisen Cell Technology Co., Ltd. (Zhejiang, China). A fibrotic induction medium was prepared by supplementing DMEM/F12 complete medium (Zhongqiao Xinzhou Biotechnology Co., Ltd., Shanghai, China) with transforming growth factor-β1 (TGF-β1) at a final concentration of 20 ng/mL. IHESC-SV40 cells were seeded in 6-well plates at a density of 2 × 10^5^ cells per well and treated with the induction medium for 24 h. The successful establishment of the fibrotic cell model was confirmed by measuring the mRNA expression levels of fibrosis-related marker genes (COL1A1, COL3A1, ACTA2, FN1).

### Quantitative real-time polymerase chain reaction (qRT-PCR)

Total RNA was extracted from cells using a Total RNA Extraction Kit (NCM Biotech, Suzhou, China) according to the manufacturer’s instructions. Complementary DNA (cDNA) was synthesized using the ToloScript All-in-One RT Easy Mix for qPCR with the following reaction conditions: 25 °C for 5 min, 60 °C for 15 min, and 85 °C for 5 s. Quantitative real-time PCR (qPCR) was performed on an ABI QuantStudio 3 Real-Time PCR System under the following cycling conditions: initial denaturation at 95 °C for 30 s, followed by 40 cycles of 95 °C for 10 s and 60 °C for 30 s. Relative mRNA expression levels were calculated using the 2^−^ΔΔCt method, with GAPDH used as the internal reference gene. All experiments were repeated at least 3 times.

### Western blot (WB)

Total protein was extracted from tissues and cells with RIPA lysis buffer (P0013B, Beyotime) supplemented with Protein Phosphatase Inhibitor (P1260, Solarbio) and quantified using the Enhanced BCA Protein Assay Kit (P0010, Beyotime). Proteins were separated by 10% SDS-PAGE (P0212, NCM Biotech) and transferred onto polyvinylidene difluoride membranes (FFP39, Beyotime). After blocking with NcmBlot Blocking Buffer 10 min (P30500, NCM Biotech), the membranes were incubated in primary antibody overnight at 4 °C.

The following primary antibodies were used: ActivAb™ Anti-ACTA2 Polyclonal Antibody (K109667P, dilution 1:1000, Solarbio), Fibronectin Polyclonal Antibody (15613-1-AP, dilution 1:1000, Proteintech), *Transgelin* Monoclonal Antibody (10493-1-AP, dilution 1:1000, Proteintech), TNS1 Polyclonal antibody (20054-1-AP, dilution 1:500, Proteintech), β-Tubulin Rabbit Monoclonal Antibody (AF1216, dilution 1:1000, Beyotime), Collagen Type I Monoclonal Antibody (67288-1-Ig, dilution 1:1000, proteintech), Collagen Type III (N-terminal) Polyclonal Antibody (22734-1-AP, dilution 1:1000, proteintech). After overnight incubation at 4 °C, membranes were washed 3 times with 1×TBST (WB20500, NCM Biotech). Secondary antibodies AF488-labeled Goat Anti-Rabbit IgG (H + L) (A0423, dilution 1:20,000, Beyotime) were applied to incubation at room temperature for 60 min. The expression of the specific protein was detected using the Li-Cor Odyssey 9120 Imaging System, with signals acquired at 800 nm.

### Collagen gel contraction assay

The assay was performed using the Cell Contraction Assay Kit (CBA-201, Cellbiolabs, Beijing, China). Collagen working solution A was prepared according to the manufacturer’s instructions. Cell suspension was prepared in DMEM/F12 basal medium at 2 × 10^5^ cells/mL. Solution A was mixed with the cell suspension at a 4:1 ratio. A total of 500 μL of the mixture was added to each well of a 24-well plate and incubated at 37 °C with 5% CO2 for 1 h to allow collagen polymerization. After polymerization, 1 mL of DMEM/F12 medium was added, and the gel was cultured for 48 h. The collagen gel was gently released from the well wall using a sterile scraper. Images of the gel were captured at different time points (4, 8, and 12 h), and the gel area was quantified using ImageJ software.

### Flowcytometry

ESMCs were resuspended in DMEM/F12 complete medium, and 1 × 10^5^ cells were collected for intracellular flow cytometry analysis. Cells were fixed with 4% paraformaldehyde for 15 min at 24 °C temperature, followed by permeabilization methanol at −20 °C for 4 h. After washing with flow cytometry buffer, cells were incubated overnight at 4 °C with the following primary antibodies: TNS1 polyclonal antibody (20054-1-AP, dilution 1:500, Proteintech), *transgelin*/SM22 monoclonal antibody (60213-1-Ig, dilution 1:1000, Proteintech), and *transgelin* monoclonal antibody (10493-1-AP, 1:1000, Proteintech).

Subsequently, intracellular staining was performed using fluorophore-conjugated secondary antibodies, including FITC-labeled goat anti-rabbit IgG (A0562, dilution 1:1000, Beyotime) and Cy3-labeled goat anti-mouse IgG (A0521, dilution 1:1000, Beyotime), for 30 min at 4 °C in the dark. Cells were washed with flow cytometry buffer between each step. Data acquisition was performed using a calibrated BD FACSCanto II flow cytometer, and fluorescence signals were recorded using BD FACSDiva™ software (v8.0.1). Data analysis was conducted using FlowJo software (v10.8.1). The proportions of TAGLN-positive, TNS1-positive, and TAGLN/TNS1 double-positive cells were quantified. Positive populations were defined based on the fluorescence background of the secondary antibody-only control. Data are presented as the percentage of HESCs exhibiting high co-expression of TAGLN and TNS1.

### Measurement of intracellular and mitochondrial reactive oxygen species

Intracellular reactive oxygen species (ROS) levels were measured using a ROS Detection Kit (Beyotime, China), while mitochondrial superoxide production was assessed using a mitochondrial superoxide detection kit (MitoSO™ Red). After removal of the culture medium, cells were incubated with either DCFH-DA or MitoSO™ Red working solution (1 μL probe diluted in 1 mL serum-free medium) at 37 °C in a humidified incubator with 5% CO2 for 30 min. For detection of mitochondrial superoxide levels, cells stained nuclei with Hoechst 33,342 solution (1 μL probe diluted in 1 mL serum-free medium) for 10 min. Cells then incubated mitochondria with MitoTracker Green working solution (C1048, Beyotime; 1 μL probe diluted in 1 mL serum-free medium) for 30 min. Following incubation, the probe-containing medium was discarded, and cells were washed three times with ice-cold phosphate-buffered saline (PBS).

Fluorescence signals were initially visualized using a fluorescence microscope. Cells were then harvested using a cell scraper, resuspended in 300 μL PBS, and subjected to flow cytometric analysis. Intracellular ROS and mitochondrial superoxide levels were quantified based on the percentage of positive cells and the mean fluorescence intensity (MFI) detected in the FITC-H and PE-H channels.

### Rat intrauterine adhesion model establishment

Adult female Sprague-Dawley (SD) rats (8 weeks old) were purchased from Jilin Ark Animal Breeding Farmers’ Cooperative (Jilin, China). Animals were randomly assigned to the IUA group or the sham-operated group. Vaginal smears were collected daily and examined to determine the estrous cycle stage. Only rats in estrus were used for surgical procedures. All animal experimental procedures were reviewed and approved by the Experimental Animal Ethics Committee of the Basic Medical College, Jilin University (Approval No. 2025-YS-736), and were conducted in accordance with the National Institutes of Health Guide for the Care and Use of Laboratory Animals.

Rats were anesthetized with isoflurane inhalation and placed in the supine position. After abdominal hair removal, the surgical area was disinfected three times with povidone–iodine, and a vertical midline incision (∼3 cm) was made in the lower abdomen to expose the uterus. In the IUA group, the uterine cavity was mechanically injured by scraping the endometrial surface using a sterile blunt metal needle pre-soaked in lipopolysaccharide (LPS, 5 μg/mL), with 20 strokes applied to each uterine wall. The uterus was then repositioned, and the abdominal incision was closed in layers. The sham-operated group underwent the same surgical exposure and incision closure without endometrial injury. All surgical procedures were performed according to methods previously described by Xu et al. (2022) Rats were euthanized in batches during estrus at 2, 4, 6, and 12 weeks after surgery for subsequent analyses.

### Collection and isolation of endometrial stromal cells

The uterine horn on the modeled side presented with bending and curling. Under sterile conditions, bilateral uterine horns were excised and placed in phosphate-buffered saline (PBS). Adipose and connective tissues surrounding the uterus were carefully removed using forceps and scissors. Each uterine horn was opened longitudinally along its long axis to expose the endometrium. The tissues were rinsed repeatedly with PBS to remove blood and mucus. The endometrial layer (pinkish and villus-like) was gently scraped off using forceps, with minimal contamination of the myometrium. The scraped endometrial tissue was minced into approximately 1 mm^3^ fragments. The minced tissue was digested with 1% type I collagenase. Briefly, tissue fragments were placed in a centrifuge tube and mixed with five volumes of collagenase solution. Digestion was performed in a 37 °C water bath or incubator with shaking (approximately 100 rpm) for 60 min. Every 20 min, the suspension was triturated with a pipette to facilitate cell dissociation. At the end of digestion, an equal volume of culture medium supplemented with 10% fetal bovine serum (FBS) was added to stop the enzymatic reaction. The digested cell suspension was filtered through a cell strainer with a pore size of approximately 100 μm. The filtrate was collected and centrifuged at 1000 rpm for 10 min at room temperature. The supernatant was discarded, and the cell pellet was resuspended in DMEM/F12 medium containing 10% FBS. The cells were seeded in culture flasks or dishes and incubated at 37 °C in a 5% CO_2_ atmosphere. After 30 min of incubation, the flasks were gently swirled, and the non-adherent cells were aspirated. The adherent cells were washed three times with PBS. Subsequently, 0.25% trypsin-EDTA solution was added to cover the cell layer and incubated for 2 min at 37 °C. Cell rounding and detachment were monitored under a microscope. Immediately when cells began to detach, an equal volume of complete medium containing 10% FBS was added to terminate trypsinization. The culture flask bottom was gently pipetted to collect the detached cells, and the flask bottom was rinsed once with PBS. The cell suspensions were combined and centrifuged at 1000 rpm for 5 min at room temperature. After discarding the supernatant, the cell pellet was resuspended in 1–2 mL of PBS. A 10 μL aliquot was taken for cell counting using a hemocytometer (Yang et al., 2024). The resulting cell suspension was used for subsequent Western blot analysis.

### Hematoxylin–eosin stain

Uterine tissues were immediately fixed in 10% neutral-buffered formalin for 24 h and subsequently dehydrated through a graded ethanol series. After clearing in xylene, tissues were embedded in paraffin. Paraffin blocks were sectioned, deparaffinized, rehydrated, and stained using hematoxylin and eosin solutions (Servicebio, China). Stained slides were examined under a light microscope to evaluate histopathological changes, including glandular number and morphology, and to assess the severity of IUA formation.

### Masson’s trichrome stain

Paraffin-embedded sections were deparaffinized, rehydrated, and stained using a Masson’s trichrome staining kit (Solarbio, China). Collagen fibers were identified by their characteristic blue staining. Quantitative analysis of collagen deposition was performed using ImageJ software (NIH, United States), and the collagen-positive area was calculated as a percentage of the total tissue area.

### Immunohistochemistry

Paraffin-embedded tissue sections were deparaffinized and rehydrated.Immunohistochemical staining was performed for TNS1 and TAGLN antibodies (20054-1-AP and 60213-1-Ig). Following antigen retrieval, endogenous peroxidase activity was quenched by incubating the sections in 3% hydrogen peroxide, and nonspecific binding was blocked with normal serum. The sections were then incubated with primary antibodies for 24 h, followed by biotinylated secondary antibodies and streptavidin–horseradish peroxidase complex, and visualized using a chromogenic substrate. Nuclei were counterstained with hematoxylin, and the sections were mounted.

### Immunofluorescence

Paraffin-embedded skin tissue sections were deparaffinized in xylene and rehydrated through a graded ethanol series to distilled water. Antigen retrieval was performed by incubating the sections in sodium citrate buffer (pH 7.5) in a 100 °C water bath for 30 min. After retrieval, the sections were rinsed in distilled water for 10 min to remove residual retrieval solution. The sections were then blocked with 5% (w/v) nonfat dry milk in PBS at room temperature for 20 min to reduce non-specific antibody binding. After blocking, the sections were rinsed with PBS. According to the experimental design, working solutions of primary antibodies against TAGLN and TNS1 were evenly applied onto the tissue sections. The sections were incubated in a humidified chamber at 4 °C overnight to allow sufficient binding of the antibodies to their target antigens. On the following day, the primary antibody solution was removed, and the sections were washed twice with TBST and once with PBS (total wash time: 10 min) to remove unbound primary antibodies. Subsequently, fluorophore-conjugated secondary antibodies (1:200 dilution) were applied, and the sections were incubated at 37 °C for 1 h in the dark. An Alexa Fluor 488-conjugated secondary antibody was used for green fluorescence detection to label TAGLN, and an Alexa Fluor 594-conjugated secondary antibody was used for red fluorescence detection to label TNS1. After secondary antibody incubation, the sections were again washed twice with TBST and once with PBS (total wash time: 10 min) to remove unbound secondary antibodies. Finally, the sections were mounted with an anti-fade mounting medium. After mounting, the stained sections were examined and imaged under a fluorescence microscope. The expression patterns and subcellular localization of the target proteins within the tissue were recorded.

### Statistical analysis

Bioinformatic analysis were performed using R software (version 4.3.2) with the built-in statistical methods provided by the corresponding R packages. Statistical analyses *in Vitro* and *Vivo* experiments were conducted using GraphPad Prism 10.4.1 (GraphPad Software, San Diego, CA, United States). Study groups were summarized using descriptive statistics appropriate to the distribution of the variables. Differences between two groups were assessed using Student’s t-test. Temporal changes in TNS1 and TAGLN expression in IHC analysis utilized one-way ANOVA with Tukey’s post hoc correction. *p* < 0.05 was considered statistically significant. Data are presented as mean ± standard deviation (SD) or as median with range.

## Results

### Integrated transcriptomic analyses identify TNS1 and TAGLN as key genes associated with IUA

**FIGURE 2:**
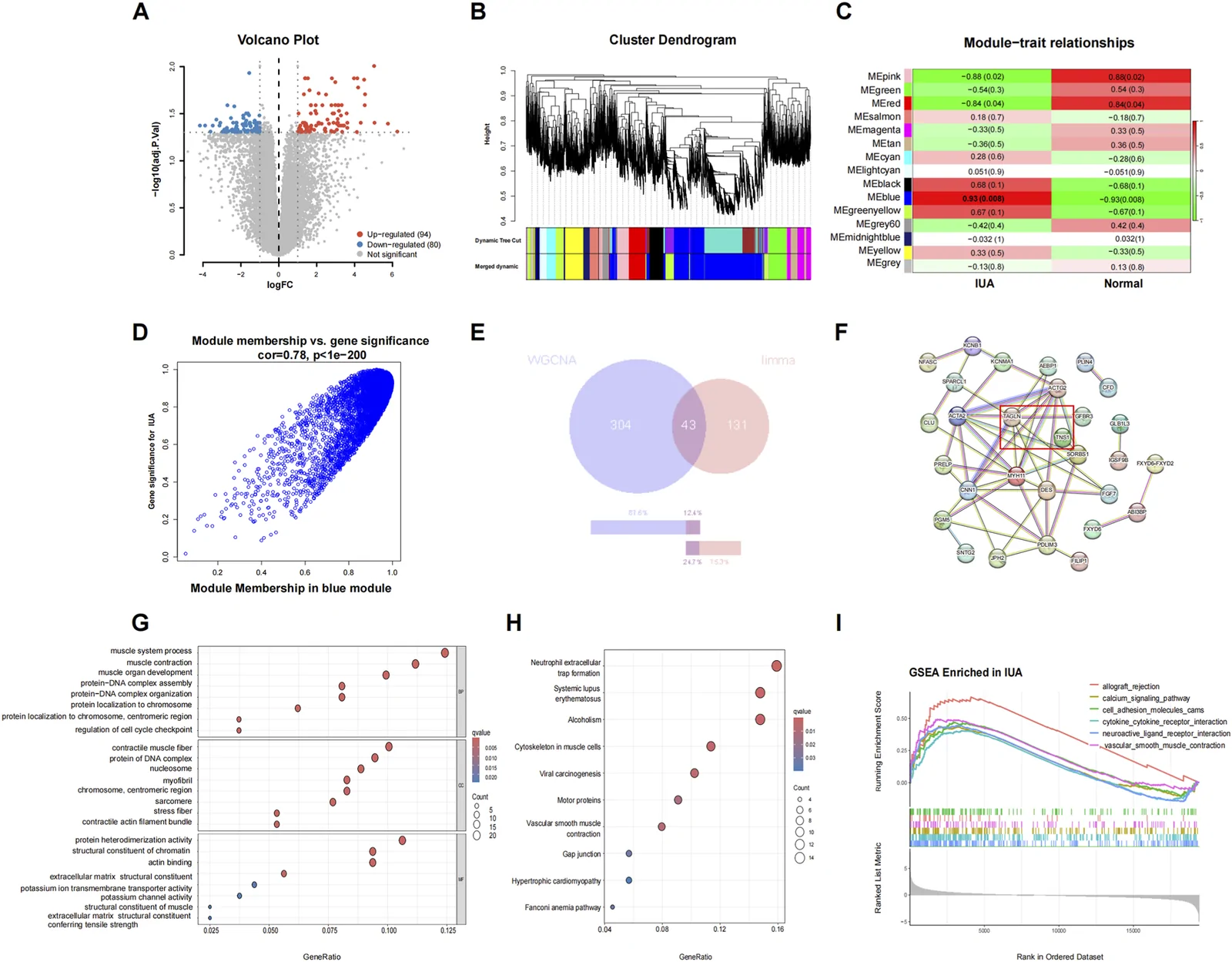
Identified TNS1 and TAGLN as key genes in IUA. **(A)** Differentially expressed genes in GSE215968 shown as Volcano plot. **(B–D)** Identification of IUA-related key gene modules using weighted gene co-expression network analysis (WGCNA). **(E)** Venn diagram showing the intersection of key genes, yielding 42 candidate genes. **(F)** Protein–protein interaction (PPI) network of the identified key genes. **(G)** Gene Ontology (GO) functional enrichment analysis, including biological process (BP), cellular component (CC), and molecular function (MF). **(H)** Kyoto Encyclopedia of Genes and Genomes (KEGG) pathway enrichment analysis of DEGs. **(I)** Gene set enrichment analysis (GSEA).

To identify key genes involved in the pathogenesis of IUA, differential gene expression analysis was performed using the LIMMA algorithm on bulk endometrial transcriptomic data from IUA and control samples. A set of significantly differentially expressed genes (DEGs) was identified, as visualized in the volcano plot, revealing both upregulated and downregulated genes in IUA tissues (Figure 2A). Weighted gene co-expression network analysis (WGCNA) was subsequently conducted to explore coordinated gene expression patterns associated with IUA. Several gene modules were identified, among which one module exhibited the strongest correlation with the IUA phenotype (Figures 2B,C). Genes within this module displayed high module membership and gene significance, suggesting close relevance to disease progression (Figure 2D).

Intersection analysis between LIMMA-derived DEGs and genes within the IUA-associated WGCNA module identified a subset of overlapping genes. Among these, TNS1 and TAGLN consistently emerged as key candidates (Figures 2E,F). Functional enrichment analyses revealed that these genes were significantly enriched in pathways related to cytoskeletal organization, smooth muscle contraction, ECM remodeling, and cell adhesion—processes closely linked to fibrotic remodeling of the endometrium (Figures 2G-I). These findings suggest that TNS1 and TAGLN are strongly associated with IUA-related pathological changes.

### Single-cell RNA sequencing reveals stromal cell–biased expression of TNS1 and TAGLN in IUA

To further characterize the cell type–specific distribution of TNS1 and TAGLN, we analyzed single-cell RNA sequencing (scRNA-seq) data derived from endometrial tissues of patients with IUA and matched controls. Using the Seurat package (version 4.4.0), we performed unsupervised clustering followed by visualization with uniform manifold approximation and projection (UMAP). Cell clusters were annotated based on SingleR analysis in combination with reference information from the CellMarker database.

**FIGURE 3:**
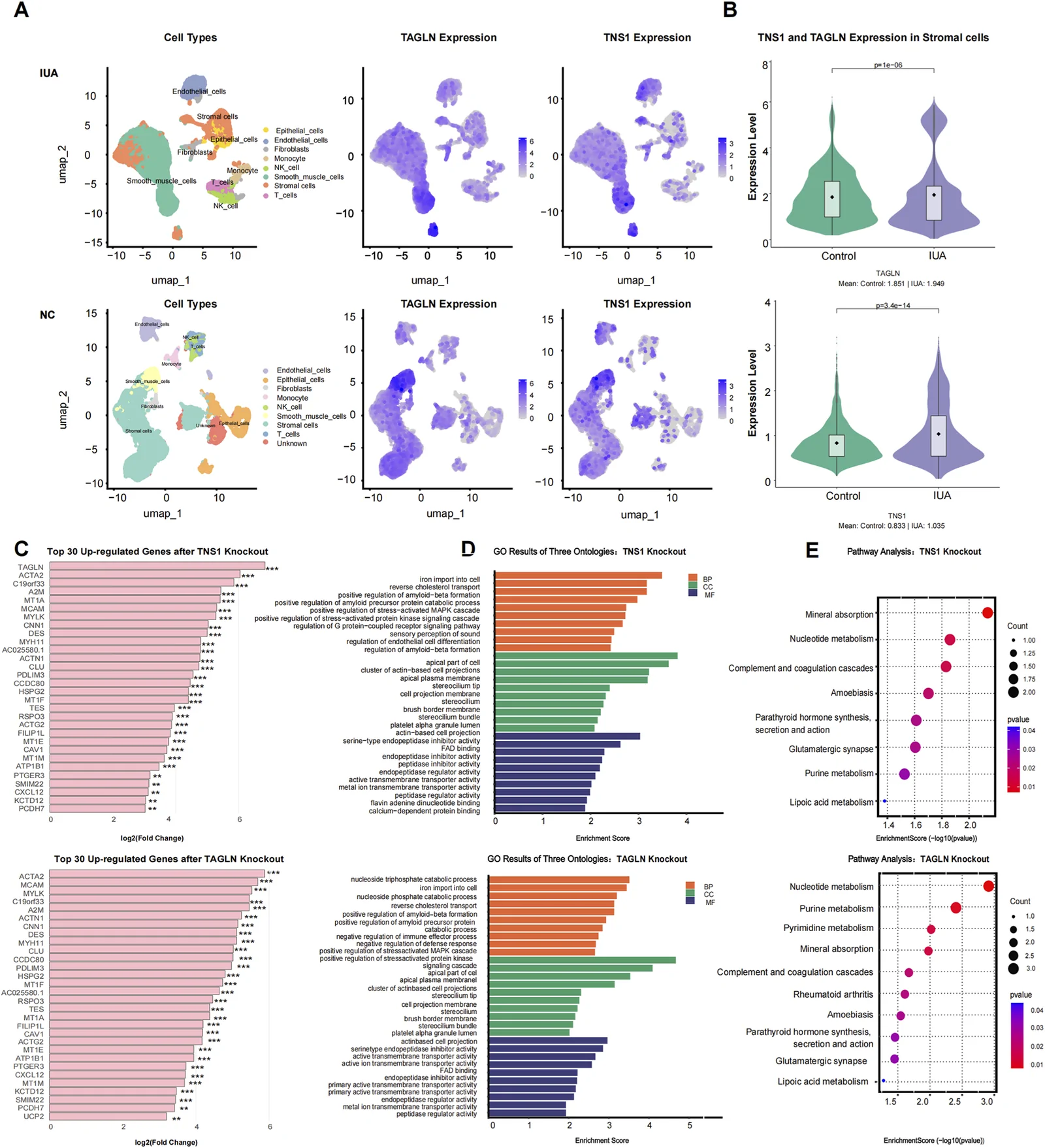
Cell-type specific expression and functional relevance of TNS1 and TAGLN in IUA. **(A)** UMAP plots showing the expression of TNS1 and TAGLN across major cell types in the GSE215968. **(B)** Violin plots comparing TNS1 and TAGLN expression levels in endometrial stromal cells between normal and intrauterine adhesion samples. **(C)** Top 30 differentially expressed genes following virtual knockdown of TNS1 or TAGLN in stromal cells. **(D)** Gene Ontology (GO) enrichment analysis of differentially expressed genes after virtual knockdown of TNS1 or TAGLN. **(E)** KEGG pathway enrichment analysis of differentially expressed genes after virtual knockdown of TNS1 or TAGLN.

A total of 18 clusters were identified and subsequently classified into seven major cell types, including epithelial cells, endothelial cells, fibroblasts, stromal cells, smooth muscle cells, monocytes, natural killer (NK) cells, and T cells. Feature plots demonstrated that TNS1 and TAGLN were expressed across multiple cell populations, including smooth muscle cells, epithelial cells, endothelial cells, fibroblasts, and stromal cells (Figure 3A).

Violin plot visualization combined with statistical comparison revealed that, compared with control samples, stromal cells from IUA tissues exhibited significantly higher expression levels of both TNS1 and TAGLN (*p* < 0.05; Figure 3B), indicating stromal cell–biased upregulation of these genes in the disease context.

To explore the functional relevance of TNS1 and TAGLN in stromal cells, we performed *in silico* knockdown of TNS1 and TAGLN in HESCs. Figure 3C summarizes the top 30 genes showing altered expression following gene knockdown. Notably, virtual knockdown of TNS1 was accompanied by a potential compensatory increase in TAGLN expression. A complete list of differentially expressed genes is provided in Supplementary Table S1.

Gene Ontology (GO) and Kyoto Encyclopedia of Genes and Genomes (KEGG) enrichment analyses were then conducted using all genes with altered expression following TNS1 or TAGLN knockdown. The enrichment profiles showed a high degree of overlap between the two perturbations (Figures 3D,E). Following TNS1 knockdown, differentially expressed genes were significantly enriched in terms related to adhesive and structural components, including apical plasma membrane and cell projection membrane, as well as pathways associated with epithelial differentiation and regulation of G protein–coupled receptor (GPCR) signaling. In contrast, TAGLN knockdown led to broad enrichment of metabolic pathways, including purine, pyrimidine, nucleotide, and lipoic acid metabolism, along with pathways associated with negative regulation of immune and defense responses and chronic inflammatory–fibrotic processes characteristic of rheumatoid arthritis.

Importantly, both TNS1 and TAGLN knockdown shared enrichment in the positive regulation of the stress-activated MAPK cascade. Collectively, these findings indicate that stromal cells in IUA display disease-associated transcriptional programs characterized by coordinated regulation of TNS1- and TAGLN-related pathways.

### Increased TNS1^+^TAGLN^+^ stromal cell subsets associates with a fibrotic state of HESCs in IUA

To confirm the successful establishment of the IUA model in HESCs, we first examined the expression of fibrosis-related genes at the mRNA level. Quantitative analysis showed that COL1A1, FN1, and ACTA2 were significantly upregulated, whereas COL3A1 expression was reduced in fibrotic HESCs. Consistently, Western blot analysis demonstrated increased protein levels of fibronectin, α-smooth muscle actin (α-SMA) and COL1A1, while the COL3A1 was reduced in fibrotic HESCs. These results confirm the successful induction of a fibrotic IUA-like phenotype in HESCs.

**FIGURE 4:**
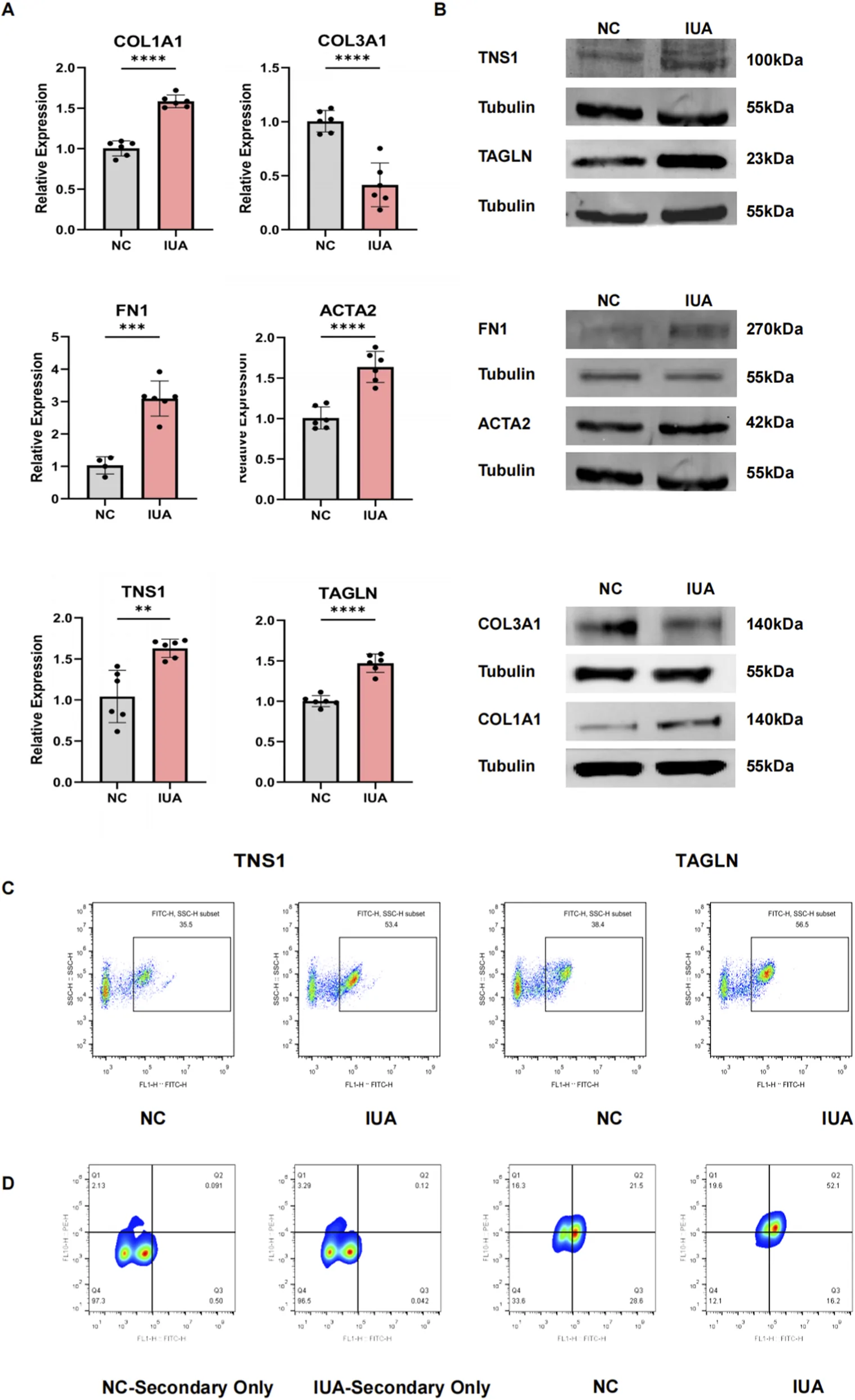
Upregulation of TNS1 and TAGLN is associated with enhanced fibrotic features in intrauterine adhesion. **(A)** mRNA expression of COL1A1, COL3A1, FN1, ACTA2, TNS1 and TAGLN. Data are presented as mean ± SD (n = 6). *P* < 0.05 (*), *P* < 0.01 (**), *P* < 0.001 (***)*P* < 0.0001 (****). **(B)** Protein expression of TNS1 and TAGLN, Protein expression of FN1 and ACTA2,Protein expression of COL1A1 and COL3A1. **(C)** Flow cytometric analysis of TNS1 and TAGLN positive cells (FITC-labeled) in NC and IUA groups. **(D)** Dual-color flow cytometric analysis of TNS1 and TAGLN expression. Cells from NC and IUA groups were analyzed by dual-color flow cytometry. TNS1 was detected in the FITC-H and TAGLN in the PE-H. Secondary antibody–only controls were used to assess nonspecific binding and to establish gating boundaries.

We next assessed the expression of TNS1 and TAGLN in the cellular model at both the transcriptional and protein levels. Compared with normal HESCs, fibrotic HESCs exhibited significantly elevated expression of TNS1 and TAGLN at both mRNA and protein levels. Flow cytometric analysis further supported these findings (Figures 4A,B).

To further determine whether TNS1/TAGLN-high stromal cells represent a specific disease-associated stromal cell state in IUA rather than a fixed stromal lineage, we performed additional validation using flow cytometry. In the IUA model group, the proportion of TNS1^+^TAGLN^+^ HESCs increased from 21.5% to 52.1%, a result that closely aligned with the findings obtained from single-marker staining of TNS1 or TAGLN (Figures 4C,D). These data indicate the emergence of a disease-associated fibrotic state of HESCs, rather than the expansion of a distinct stromal cell lineage.

### Enhanced contractility and oxidative stress characterize stromal cells in the IUA *vitro* model

**FIGURE 5:**
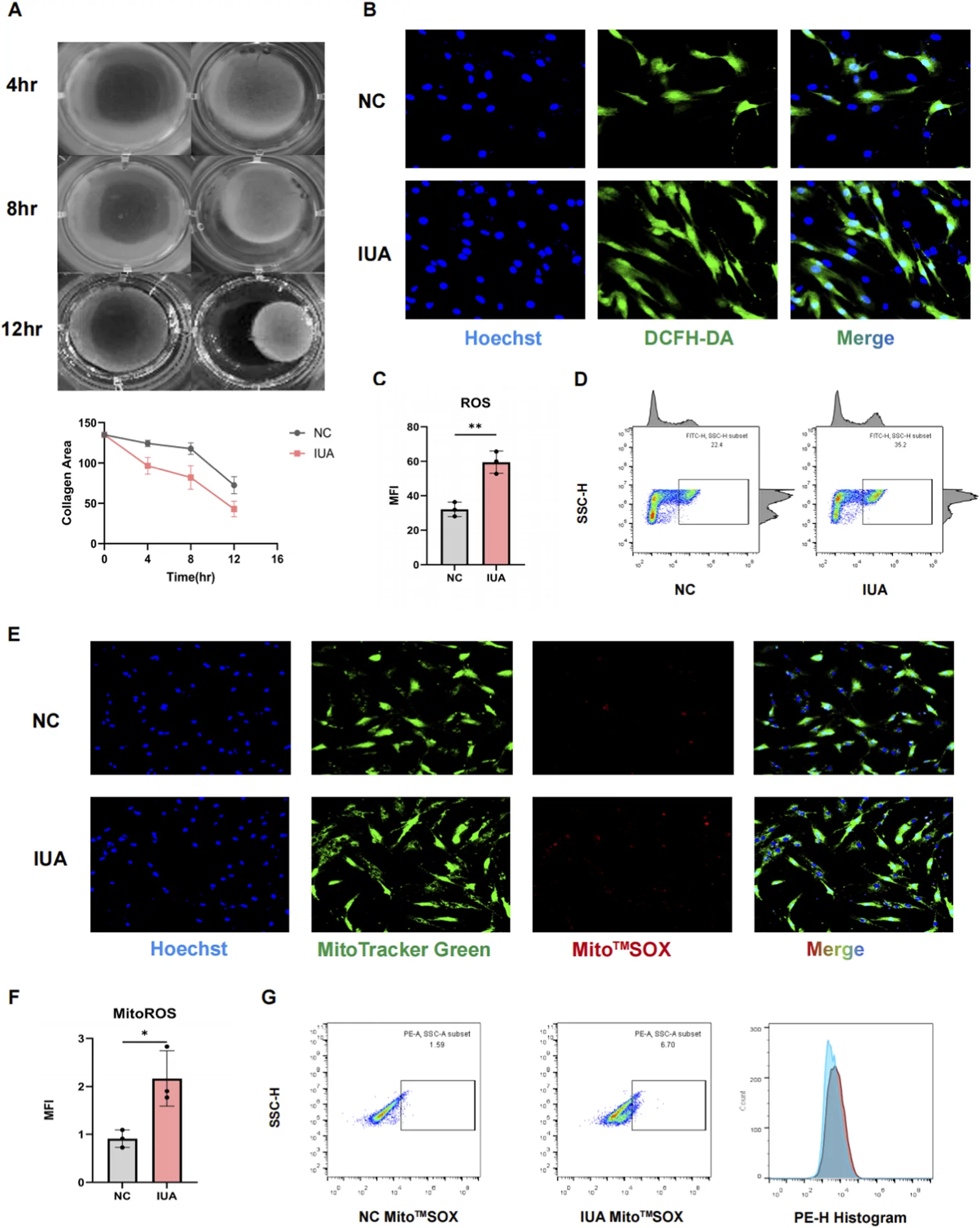
Increased collagen contraction and oxidative stress in IUA-derived cells. **(A)** Representative images and quantitative analysis of collagen gel contraction in NC and IUA cells at the indicated time points (n = 4). **(B)** Representative fluorescence images of intracellular ROS detected by DCFH-DA staining; nuclei were counterstained with Hoechst. **(C)** Quantification of intracellular ROS levels expressed as MFI. (n = 3). **(D)** Flow cytometric analysis of intracellular ROS levels in NC and IUA. **(E)** Representative fluorescence images of mitochondrial superoxide detected by MitoTMSOX staining; mitochondria were labeled with MitoTracker Green and nuclei with Hoechst33342. **(F)** Quantification of mitochondrial superoxide levels expressed as MFI (n = 3). **(G)** Flow cytometric analysis of mitochondrial superoxide levels in NC and IUA. Data are presented as mean ± SD. *P* < 0.05 (*), *P* < 0.01 (**), *P* < 0.001 (***)*P* < 0.0001 (****).

Given the close involvement of TNS1 and TAGLN in cytoskeletal regulation, we next evaluated the functional characteristics of HESCs in the IUA model. Collagen gel contraction assays demonstrated that cells in the IUA model exhibited a significantly enhanced contractile capacity within 48 h. Compared with normal HESCs, TGF-β1–treated cells showed an approximately 30% reduction in collagen gel area, indicating increased cellular contractility (Figure 5A).

To further assess oxidative stress levels in the IUA model, intracellular reactive oxygen species (ROS) were examined. Fluorescence microscopy revealed that the mean intracellular ROS fluorescence intensity was increased by approximately 1.5-fold in the IUA model. Consistently, flow cytometric analysis showed that the proportion of ROS-positive cells increased from 22.45% to 35.2% (Figures 5B–D).

To determine whether the elevated oxidative stress predominantly originated from mitochondria, mitochondrial oxidative stress was further evaluated. Fluorescence microscopy images demonstrated a marked increase in mitochondrial superoxide levels in the IUA model. Correspondingly, flow cytometric analysis revealed that the percentage of PE-H–positive cells was approximately 3-fold higher than that observed in normal HESCs (Figures 5E–G).

Collectively, these findings indicate that HESCs under IUA-like conditions display enhanced contractility accompanied by elevated intracellular and mitochondrial oxidative stress.

### Time-dependent upregulation of TNS1 and TAGLN in the endometrial stroma of a rat IUA model

**FIGURE 6:**
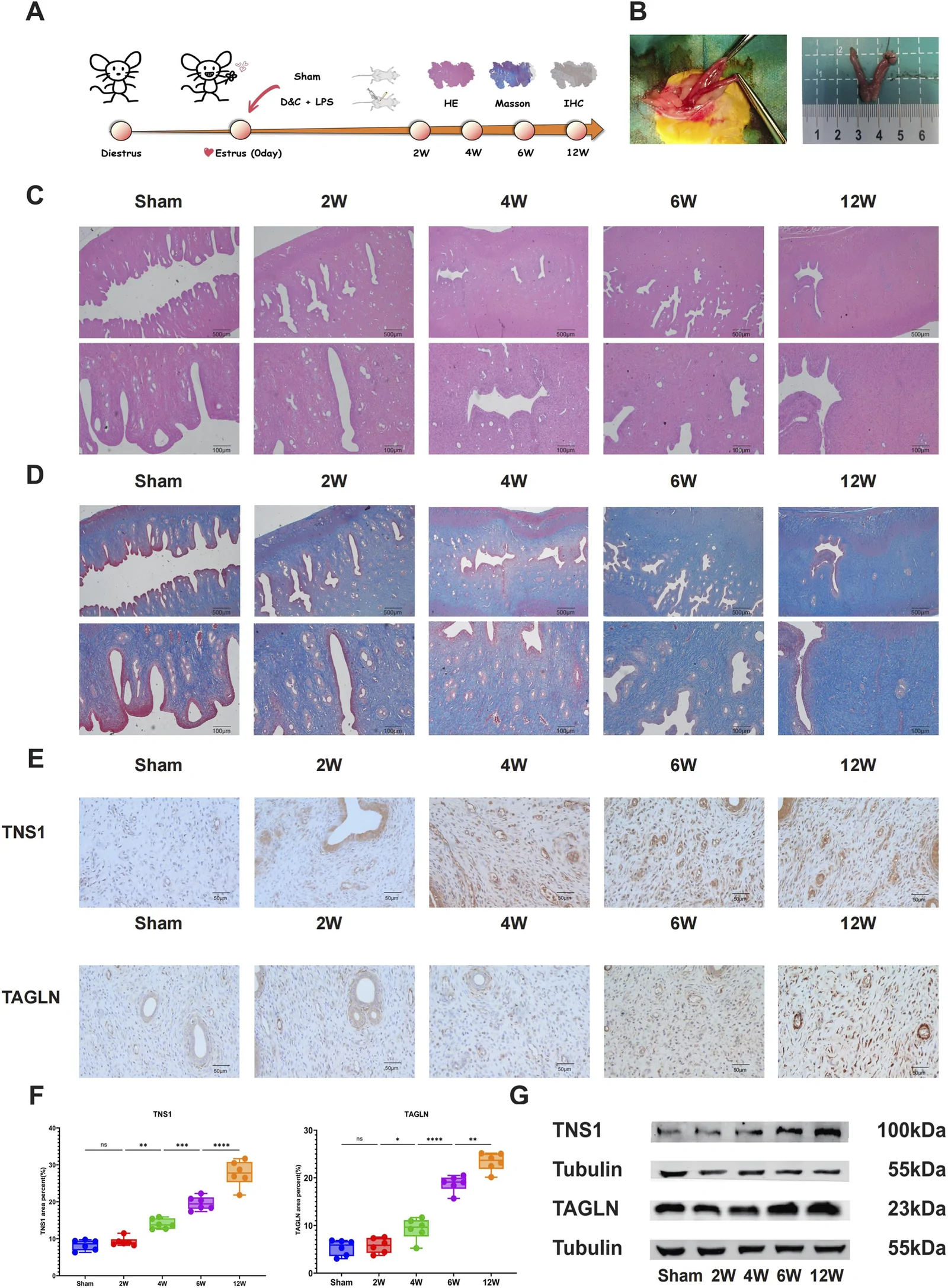
Establishment and pathological characterization of a rat IUA model. **(A)** Schematic diagram illustrating the establishment of the rat IUA model using uterine curettage combined with lipopolysaccharide (D&C + LPS) treatment and the experimental timeline. **(B)** Representative intraoperative image and gross morphology of the uterus following IUA model induction. **(C)** Representative H&E staining images of uterine tissues from the sham group and IUA model at 2, 4, 6, and 12 weeks after surgery. **(D)** Representative Masson’s trichrome staining images showing collagen deposition in uterine tissues at the indicated time points. **(E)** Representative immunohistochemical staining images of TNS1 expression in uterine tissues from sham and IUA groups at different time points. Representative immunohistochemical staining images of TAGLN expression in uterine tissues from sham and IUA groups at different time points. **(F)** Quantitative analysis of TNS1 and TAGLN positive areas based on immunohistochemical staining at different time (n = 6). Quantitative analysis of TNS1 and TAGLN positive areas based on Immunofluorescence staining at different time (n = 6). **(G)** Results of Western blot analysis of endometrial cells from the sham operation group and the experimental group at 2, 4, 6, and 12 weeks.

To further examine whether the *in vitro* findings were recapitulated *in vivo*, we established a rat model of IUA and collected uterine tissues at multiple time points following injury. Gross anatomical examination showed that, over time, the injured uterine horns developed a tortuous and irregular appearance, accompanied by the progressive formation of IUA (Figures 6A,B).

Histological analysis using hematoxylin and eosin (H&E) staining revealed that, during 2–6 weeks after injury, the number and area of endometrial glands exhibited a decreasing trend, together with disruption of the normal columnar epithelial architecture. By 12 weeks, glandular structures were markedly diminished, and the uterine cavity was largely obliterated (Figure 6C). Consistently, Masson’s trichrome staining demonstrated a progressive increase in the proportion and intensity of blue-stained areas within the uterine cavity, indicating a time-dependent accumulation of collagen in the endometrial stroma and changes consistent with progressively aggravated fibrosis (Figure 6D). Furthermore, we supplemented Masson’s trichrome staining of the sham operation group at various time points. The results indicated that collagen deposition in the endometrium of the sham operation group did not increase significantly over time (Supplementary Material 2).

For immunohistochemical analyses, quantitative assessment focused on the endometrial stromal compartment, with blank areas, myometrium, and regions of strong vascular smooth muscle staining excluded from image analysis. TNS1 showed prominent enrichment in glandular columnar epithelial cells, whereas TAGLN exhibited strong expression in the myometrium and vascular smooth muscle. Within HESCs, both TNS1 and TAGLN predominantly localized to the cytoplasm. Notably, TNS1 displayed fine linear or punctate cytoplasmic staining patterns that closely aligned with cell morphology, while TAGLN showed more pronounced staining in the periglandular stromal regions. Both proteins exhibited increased stromal expression with disease progression (Figure 6E).

**FIGURE 7:**
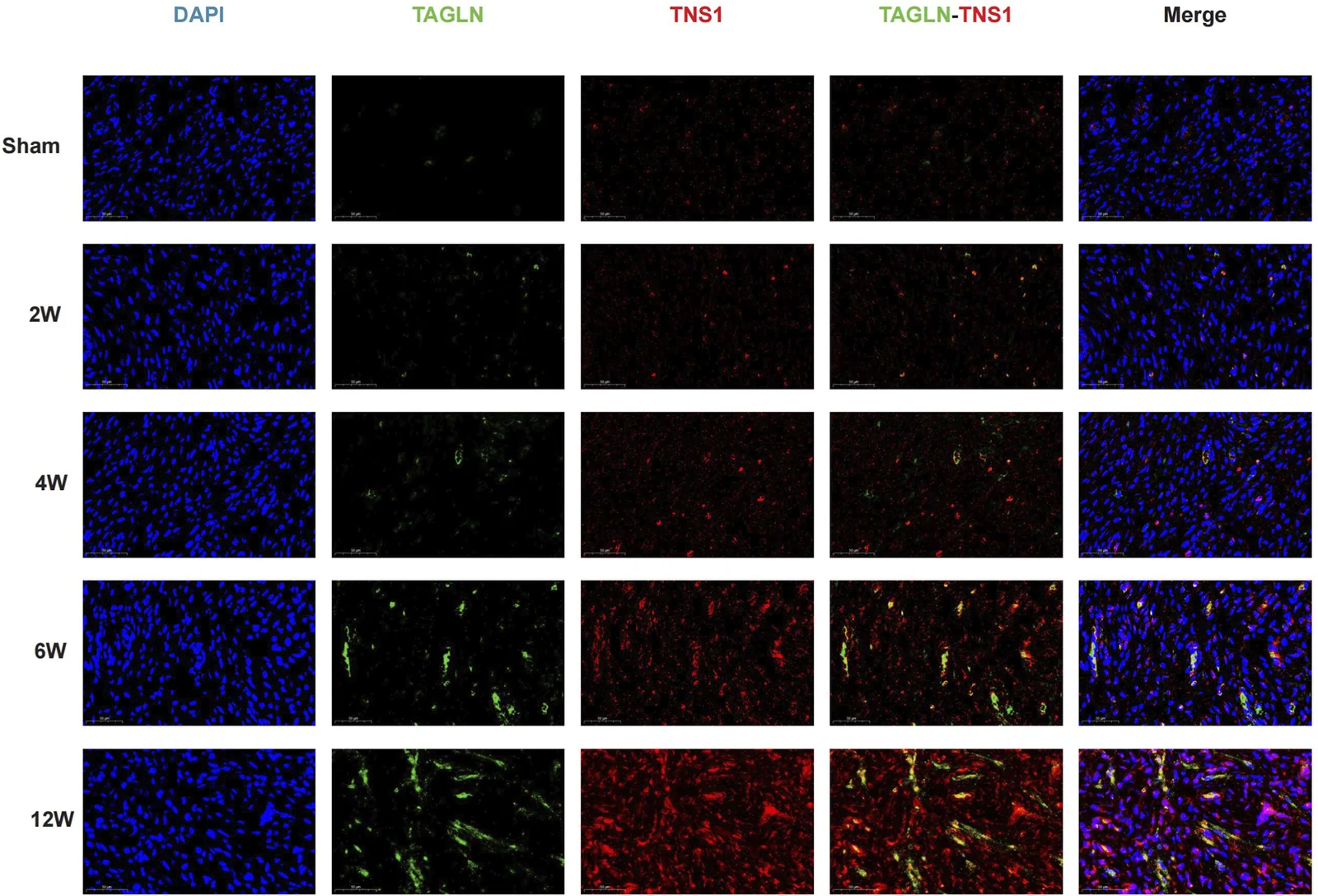
Immunofluorescence co-localization of TNS1 and TAGLN in the sham operation group and the experimental group at 2, 4, 6, and 12 weeks.

Quantitative analysis further demonstrated that changes in TNS1 expression were most pronounced during the mid-stage of injury (4–6 weeks), whereas TAGLN expression continued to increase during the later stages (6–12 weeks) (Figure 6F). We performed Western blot analysis of TNS1 and TAGLN in the endometrial cells of model rats at different time points. As shown in Figure 6G, the expression levels of TNS1 and TAGLN in the rat endometrium exhibited an increasing trend with the prolongation of modeling time. Immunofluorescence colocalization analysis revealed that the fluorescence intensity and the number of TNS1-positive signals in the non-vascular and glandular regions of the endometrial stroma progressively increased with prolonged modeling time from 4 weeks. Additionally, TAGLN expression was markedly upregulated from week 6 onward (Figure 7). Collectively, these findings indicate that TNS1 and TAGLN exhibit distinct stage-dependent expression patterns during the progression of IUA.

## Discussion

In this study, we demonstrate that IUA represent a major infertility-associated disorder in women of reproductive age and can substantially compromise menstrual patterns, embryo implantation, and pregnancy outcomes (Critchley et al., 2020). Current management relies primarily on hysteroscopic adhesiolysis combined with adjuvant hormonal therapy (Rodríguez-Eguren et al., 2024), yet postoperative recurrence remains common (Hanstede et al., 2015; Bosteels et al., 2017). Effective preventive strategies and durable treatments that reverse the underlying pathological basis of IUA is still lacking.

During the normal menstrual cycle, the endometrium undergoes cyclic remodeling coordinated by multiple cell types, characterized by a highly regulated, scar-free regenerative pattern (Jain et al., 2022; Ang et al., 2023). This process depends on precise control of cellular functional states. HESCs secrete collagens, laminin, fibronectin, proteoglycans, and forming a fibrous scaffold that support endometrial structure and plays a critical role in normal endometrial repair (Zhu et al., 2019). In recent years, single-cell transcriptomic studies of IUA have revealed heterogeneity among different endometrial cell populations and consistently emphasized the central role of HESCs in disease development (Xin et al., 2024; Deryabin and Borodkina, 2025; Lv et al., 2023; Santamaria et al., 2023; Zhang and Hu, 2024; Pardo-Figuerez et al., 2025; Guo et al., 2022).

Integrating bulk RNA sequencing and single-cell transcriptomic data, we identified TNS1 and TAGLN as key regulatory nodes in HESCs associated with IUA. We further validated these findings at both the mRNA and protein levels. Flow cytometric analysis showed an increased proportion of TNS1^+^TAGLN^+^ cells in the model group. These results suggest that this cell population with distinct pathological characteristics may be closely associated with accelerated disease progression and the maintenance of a pro-fibrotic microenvironment.

*Tensin* 1 (TNS1) mediates the formation of mature fibrillar adhesions. By directly binding to the membrane-proximal NPxY motif within the cytoplasmic tail of β1 integrin through its phosphotyrosine-binding (PTB) domain (McCleverty et al., 2007). Through this interaction, TNS1 couples integrin activation to cytoskeletal tension and the generation of traction forces, thereby promoting fibrillar assembly of fibronectin and strengthening cell–matrix interactions (Lemmon et al., 2009). In contrast, TAGLN is a calcium-dependent actin-binding protein that functions as a key effector of actomyosin contraction and is widely recognized as an important marker of activated smooth muscle cells and myofibroblasts (Cevallos et al., 2006; Sun et al., 2023).

Previous studies have shown that loss of TNS1 disrupts focal adhesion signaling, actin stress fiber formation, and fibroblast activation in pulmonary fibrosis (Bernau et al., 2017). However, TGF-β induced upregulation of TAGLN promotes the transition of quiescent fibroblasts into highly contractile myofibroblasts (Muraoka et al., 2023). Across multiple fibrosis-related diseases, including endometriosis-associated adhesions and hypertrophic skin scars, studies have identified fibroblast populations with high TAGLN expression (Cheng et al., 2025). Suppressing TAGLN expression markedly reduces collagen production, cell migratory capacity, and ECM stability (Wei et al., 2021).

Collectively, this evidence supports that TNS1 and TAGLN function at distinct yet interconnected regulatory levels within fibrotic programs, coordinating adhesion-mediated force transmission and sustained contractile execution, respectively.

Based on observations, we performed virtual knockout analyses in HESCs of single-cell data. Virtual knockout of TNS1 primarily altered the expression of genes involved in ion transport, calcium-dependent processes, and cell adhesion. In contrast, virtual knockout of TAGLN induced broader systemic changes, including marked perturbations in nucleotide metabolism, lipid and cholesterol transport, and inflammation-related pathways, together with downregulation of ATP catabolic processes and immune defense responses.

Notably, under conditions of TNS1 knockout, TAGLN expression showed compensatory upregulation, suggesting that when adhesion-related signaling becomes disrupted, HESCs may engage alternative contractile and metabolic programs to maintain a pro-fibrotic state. This compensatory mechanism may partially explain why single-node interventions targeting the cell adhesion or focal adhesion signaling axis prove insufficient to fully interrupt pro-fibrotic programs. Even when adhesion signaling is perturbed, stromal cells may sustain IUA progression through parallel compensatory activation of contractile and metabolic pathways (Bernau et al., 2017).

We observed that at both the mRNA and protein levels, COL1A1 expression was upregulated in the cell model of IUA, whereas COL3A1 expression was higher in the healthy control group. Previous studies have indicated excessive type I collagen deposition in adenomyotic lesions, while type III collagen showed no significant increase compared with healthy control tissues (Kishi et al., 2017). A similar trend has been observed in cardiac scar repair after injury, cutaneous wound healing (Yeung and Kelly, 2021), and experimentally induced myoblast fibrosis (Shi et al., 2021). Therefore, it is hypothesized that excessive deposition of type I collagen plays an important role in the development of intrauterine adhesions, and TNS1 and TAGLN exert a synergistic effect on type I collagen deposition.

In a rat model of IUA, we observed significant time-dependent differences in TNS1 and TAGLN expression within the endometrial stromal compartment. TNS1 showed the most pronounced changes during the mid-injury phase (4–6 weeks), whereas TAGLN expression continued to rise during the later stages (6–12 weeks). The immunofluorescence results also provide support for this speculation. This stage-specific pattern suggests an evolutionary trajectory of stromal cells transitioning from a “plastic state” to a “stable fibrotic state”. The mid-stage phase, characterized by elevated TNS1 expression, reflects a stromal cell state dominated by adhesion structure formation and mechanosensitive signaling (Wang et al., 2022; Georgiadou et al., 2017). In contrast, the sustained upregulation of TAGLN indicates a gradual shift toward a pathological state centered on contractile execution and ECM production. From a clinical perspective, this phenomenon suggests the presence of a potential “therapeutic window” in IUA. Interventions targeting adhesion remodeling and mechanical signal integration during the early or mid stages of disease may offer greater opportunities to block the transition of stromal cells toward a pro-fibrotic state. Once a fibrotic microenvironment reaches a stable equilibrium, its reversibility appears limited.

In this study, *in vitro* experiments further showed that increased expression of TNS1 and TAGLN in HESCs coincided with elevated levels of intracellular reactive oxygen species (ROS) and mitochondrial superoxide. A positive feedback loop can form between TGF-β signaling and ROS, thereby sustaining the myofibroblast phenotype and promoting ECM remodeling (Liu and Desai, 2015).

In this context, we propose that the adhesion–contraction program mediated by TNS1/TAGLN and oxidative stress may operate in a coordinated and coupled manner in IUA-associated HESCs. On the one hand, ECM stiffness and mechanical inputs regulate mitochondrial spatial positioning, fusion–fission dynamics, and metabolic functional states through integrin-associated mechanotransduction pathways (Keng et al., 2021; Crosas-Molist et al., 2023), providing a biological basis for a “mechanical signaling–mitochondrial response” axis. Adhesion-dependent mechanical signals can further modulate mitochondrial dynamics via energy-sensing pathways such as AMPK, thereby influencing mitochondrial biogenesis and oxidative metabolic capacity (Phuyal et al., 2023).

On the other hand, the establishment and maintenance of fibrosis-associated fibroblast states often coincide with metabolic reprogramming and alterations in mitochondrial function. Accumulating evidence from review studies supports a sustained and central regulatory role of mitochondria and metabolic pathways throughout fibrotic processes. Notably, a recently proposed TAGLN–RhoA/ROCK2–SLC2A3 mechano-metabolic axis in skin fibrosis further indicates that TAGLN can influence glycolytic activity and cellular motility through canonical mechanotransduction pathways, thereby coupling metabolic homeostasis beyond its role in contractile execution.

Therefore, under conditions of oxidative stress, alterations in mitochondrial structure, function, and metabolic capacity may act as an “amplifier” of the pro-fibrotic state by sustaining metabolic and contractile execution programs and reinforcing pro-fibrotic homeostasis in stromal cells.

Our study suggests that IUA may involve a distinct population of HESCs characterized by TNS1^+^TAGLN^+^ expression. Through persistently enhanced contractility, ECM remodeling capacity, and metabolic alterations, this stromal cell population may contribute to and sustain the formation of a pro-fibrotic microenvironment during disease progression. Nevertheless, several limitations remain in the present study.

First, this work primarily relies on transcriptomic data, protein expression patterns, and functional association analyses. The current evidence does not fully establish a causal regulatory framework for TNS1/TAGLN-associated signaling pathways. Future studies should integrate metabolomic profiling, direct assessments of mitochondrial function, and controllable mechanical microenvironment models to systematically elucidate the coordinated roles of mechanical signaling, metabolic homeostasis, and TNS1^+^TAGLN^+^ stromal cells in fibrotic progression.

Second, conclusions regarding stage-dependent disease evolution mainly derive from a rat IUA model. Given the substantial differences in endometrial repair dynamics and immune microenvironments across species, future investigations should incorporate human IUA samples with clearly defined disease stages to further validate the time-dependent features of stromal cell state transitions.

In addition, this study proposes a stromal cell evolutionary trajectory during IUA progression, characterized by a transition from a “plastic state” to a “stable fibrotic state,” and suggests that early or mid-stage disease may represent a potential therapeutic window. However, this hypothesis currently lacks time-resolved experimental validation. Subsequent studies could target relevant pathways at defined disease stages to evaluate their effects on fibrotic progression and endometrial repair outcomes.

## Conclusion

In summary, this study combines bulk and single-cell transcriptomic analyses with cellular and animal models to characterize stromal alterations associated with IUA. We identify consistent upregulation of TNS1 and TAGLN in endometrial stromal cells under IUA conditions and show that their increased expression accompanies fibrotic activation, enhanced contractility, and oxidative stress. In a rat IUA model, stromal expression of TNS1 and TAGLN exhibits time-dependent changes during disease progression. Collectively these findings reveal a characteristic cellular state in which endometrial stromal cells transition from physiological repair toward pathological fibrotic activation during IUA progression, and provide a cellular basis for future mechanistic and regenerative studies.

## Data Availability

The datasets presented in this study can be found in online repositories. The names of the repository/repositories and accession number(s) can be found in the article/Supplementary Material.
