# Supplementary material for: TNS1/TAGLN-enriched endometrial stromal cells is associated with a persistent fibrotic microenvironment in intrauterine adhesion
Source: Front Cell Dev Biol. 2026 Aug 13;14:1810255. doi: 10.3389/fcell.2026.1810255 (PMC13518574; doi:10.3389/fcell.2026.1810255)
Supplement: Supplementary file 1 [file Supplementaryfile6.docx]

402 [illegible] 已审 卫[illegible] 2026.1.20

吉林大学基础医学院实验动物伦理审批表

批准编号：（2025年）研审第 936 号

实验名称：亚精胺治疗子宫内膜纤维化的机制研究							
项目来源：用于预防与修复宫腔粘连的温敏型复合水凝胶的制备与机制研究							
实验目的： 通过动物实验中，优化宫腔粘连动物模型，验证亚精胺治疗与预防子宫内膜纤维化的作用							
实验动物使用许可证号：吉林大学基础医学动物实验中心 SYXK(吉)2023-0010							
申请使用实验室类别	☐普通环境设施 √☐屏障环境设施 ☐动物生物安全二级实验室<br>与许可证号一致						
项目负责人	路英丽	单位	吉林大学第二医院		联系电话	13196000879	
项目申请人	路英丽	单位	吉林大学第二医院		联系电话	13196000879	
拟使用动物情况	动物来源：长春市亿斯实验动物技术有限公司			动物品种、品系		SD 大鼠	
	动物等级	SPF	规格	200g/雌性/8-9 周龄	数量	15 只	
	麻醉途径	吸入		麻醉剂名称	异氟烷		
	处死方法： 过量麻醉/二氧化碳吸入		动物尸体和组织处理方法：按设施规定进行处理				
	实验开始日期	2025 年 12 月 3 日		实验结束日期	2026 年 2 月 7 日		

动物实验内容概述：

实验分组：实验共分 5 组，分别为“亚精胺低剂量组”“亚精胺中剂量组” “亚精胺高剂量组”“正常对照组”“单纯造模组”，每组 3 只，共 15 只。

实验内容：鉴定大鼠处于发情期，通过“机械损伤+LPS”的方法建立大鼠 IUA 模型，采用异氟烷麻醉后，充分暴露子宫，于”Y”型子宫的左侧宫体，约距子宫分叉处约 0.5cm，用大头针蘸取 10mg/L 的 LPS 溶液，在针孔处进入左侧宫腔，在宫腔前壁划 20 次，按照此流程刮其余 3 侧壁（每次均重新蘸取 LPS），后逐层关腹。手术 2 周后，过量异氟烷麻醉处死大鼠，取材子宫，通过 HE、Masson 等染色的结果和 α SMA 的表达鉴定宫腔黏连模型是否建立成功。

实验内容：IUA 模型各组大鼠分别给药剂量为：亚精胺低剂量组 50 μ mol/L ；“亚精胺—波洛沙姆”温敏水凝胶组合物；亚精胺中剂量组给予 100 μ mol/L “亚精胺—波洛沙

扫描全能王 创建

姆”温敏水凝胶组合物；亚精胺高剂量组给予 250μmol/L “亚精胺—波洛沙姆”温敏水凝胶组合物。<br>观察指标：观察宫腔粘连的情况，具体指标包括：宫腔粘连带的形成情况，子宫内膜胶原的分布情况，αSMA 的表达情况等。	
声明：我将自觉接受吉林大学基础医学院实验动物伦理委员会的监督与检查，并保证上述材料客观可靠。<br>项目负责人签字：路英丽<br>项目执行人签字：陈思瑶	
审查依据	1. 该项目是否必须用实验动物进行实验，即能否用计算机模拟、细胞培养等非生命方法替代动物或用低等动物替代高等动物进行实验？<br>2. 所用动物实验的品种品系、质量等级、规格是否合适，能否通过改良设计方案或用高质量的动物减少所用动物的数量？<br>3. 能否通过改进实验方法、调整实验观测指标、改良处死动物的方法，来优化实验方案，善待动物？
审查<br>结果	实验动物伦理委员审批意见：<br>王[illegible]<br>伦理委员会主任委员签字（盖章）：

扫描全能王 创建
