# Supplementary material for: TNS1/TAGLN-enriched endometrial stromal cells is associated with a persistent fibrotic microenvironment in intrauterine adhesion
Source: Front Cell Dev Biol. 2026 Aug 13;14:1810255. doi: 10.3389/fcell.2026.1810255 (PMC13518574; doi:10.3389/fcell.2026.1810255)
Supplement: Supplementary file 3 [file Supplementaryfile3.docx]

logFoldChange=1

adjustP=0.05

library(limma)

setwd("D:\\IUA-从头到尾\\GSE165321LIMMA+GO+KEGG+GSEA")

rt=read.table("GSE165321-RAW-MRNA-expression.txt",sep="\t",header=T,check.names=F)

rt=as.matrix(rt)

rownames(rt)=rt[,1]

exp=rt[,2:ncol(rt)]

dimnames=list(rownames(exp),colnames(exp))

rt=matrix(as.numeric(as.matrix(exp)),nrow=nrow(exp),dimnames=dimnames)

rt=avereps(rt)

rt=log2(rt+1)

#differential

modType=c(rep("IUA",3),rep("CON",3))

design <- model.matrix(~0+factor(modType))

colnames(design) <- c("treat","con")

fit <- lmFit(rt,design)

cont.matrix<-makeContrasts(con-treat,levels=design)

fit2 <- contrasts.fit(fit, cont.matrix)

fit2 <- eBayes(fit2)

allDiff=topTable(fit2,adjust='fdr',number=200000)

write.table(allDiff,file="165321A.xls",sep="\t",quote=F)

#write table

diffSig <- allDiff[with(allDiff, (abs(logFC)>logFoldChange & adj.P.Val < adjustP )), ]

write.table(diffSig,file="165321diff.xls",sep="\t",quote=F)

diffUp <- allDiff[with(allDiff, (logFC>logFoldChange & adj.P.Val < adjustP )), ]

write.table(diffUp,file="up.xls",sep="\t",quote=F)

diffDown <- allDiff[with(allDiff, (logFC<(-logFoldChange) & adj.P.Val < adjustP )), ]

write.table(diffDown,file="down.xls",sep="\t",quote=F)

#write expression level of diff gene

hmExp=rt[rownames(diffSig),]

diffExp=rbind(id=colnames(hmExp),hmExp)

write.table(diffExp,file="diffExp.txt",sep="\t",quote=F,col.names=F)

pdf(file="2.0vol165321.pdf")

\# 设置更美观的颜色

up_color <- "#E64B35"	# 红色系，代表高表达

down_color <- "#3182BD" # 蓝色系，代表低表达

base_color <- "#BDBDBD" # 灰色，代表不显著基因

\# 计算坐标范围（由于旋转，x和y轴互换）

yMax <- max(-log10(allDiff$adj.P.Val))

xMax <- max(abs(allDiff$logFC))

\# 创建旋转90°的火山图

\# 注意：x轴现在是logFC，y轴是-log10(adj.P.Val)

plot(allDiff$logFC, -log10(allDiff$adj.P.Val),

xlab = "logFC",

ylab = "-log10(adj.P.Val)",

main = "Volcano Plot ",

xlim = c(-5, 7),

ylim = c(0, yMax),

xaxs = "i",

pch = 20,

cex = 0.6,

col = base_color,

frame = FALSE)

\# 添加上调基因（高表达）

diff_up <- subset(allDiff, adj.P.Val < adjustP & logFC > logFoldChange)

points(diff_up$logFC, -log10(diff_up$adj.P.Val),

pch = 20,

col = up_color,

cex = 1.2)

\# 添加下调基因（低表达）

diff_down <- subset(allDiff, adj.P.Val < adjustP & logFC < (-logFoldChange))

points(diff_down$logFC, -log10(diff_down$adj.P.Val),

pch = 20,

col = down_color,

cex = 1.2)

\# 添加垂直线（因为旋转了，所以用v而不是h）

abline(v = 0, lty = 2, lwd = 1, col = "black")

\# 添加阈值线（可选）

abline(v = c(-logFoldChange, logFoldChange), lty = 3, lwd = 1, col = "gray50")

abline(h = -log10(adjustP), lty = 3, lwd = 1, col = "gray50")

\# 添加图例topright=右上角，bottomright=右下角

legend("bottomright",

legend = c(paste("Up-regulated (", nrow(diff_up), ")", sep = ""),

paste("Down-regulated (", nrow(diff_down), ")", sep = ""),

"Not significant"),

col = c(up_color, down_color, base_color),

pch = 20,

bty = "n",

cex = 0.7)

dev.off()

setwd("D:\\IUA-从头到尾\\WGCNA")

rt=read.table("4GSE165321-RAW-MRNA-expression.txt",sep="\t",header=T,check.names=F)

\# Load the WGCNA package

library(WGCNA)

######################################################input data##########################

\# The following setting is important, do not omit.

options(stringsAsFactors = FALSE)

#Read in the female liver data set

library(openxlsx)

fpkm=rt

#fpkm = read.table("GSE165321最终数据（codingRNA）.txt",header=T,comment.char = "",check.names=F)

##############数据文件名，根据实际修改，如果工作路径不是实际数据路径，需要添加正确的数据路径

\# Take a quick look at what is in the data set

dim(fpkm)

names(fpkm)

datExpr0 = as.data.frame(t(fpkm[,-1]))

names(datExpr0) = fpkm[,1];##########如果第一行是ID命名，就写成fpkm$ID

rownames(datExpr0) = names(fpkm[,-1])

datExpr0=log2(datExpr0+1)

##################check missing value and filter ####################

datExpr0

##check missing value

gsg = goodSamplesGenes(datExpr0, verbose = 3)

gsg$allOK

if (!gsg$allOK)

{

\# Optionally, print the gene and sample names that were removed:

if (sum(!gsg$goodGenes)>0)

printFlush(paste("Removing genes:", paste(names(datExpr0)[!gsg$goodGenes], collapse = ", ")))

if (sum(!gsg$goodSamples)>0)

printFlush(paste("Removing samples:", paste(rownames(datExpr0)[!gsg$goodSamples], collapse = ", ")))

\# Remove the offending genes and samples from the data:

datExpr0 = datExpr0[gsg$goodSamples, gsg$goodGenes]

}

##filter

meanFPKM=1 ####过滤标准，我设定的是1

n=nrow(datExpr0)

datExpr0[n+1,]=apply(datExpr0[c(1:nrow(datExpr0)),],2,mean)

datExpr0=datExpr0[1:n,datExpr0[n+1,] > meanFPKM] # for meanFpkm in row n+1 and it must be above what you set--select meanFpkm>opt$meanFpkm(by rp)

filtered_fpkm=t(datExpr0)

filtered_fpkm=data.frame(rownames(filtered_fpkm),filtered_fpkm)

names(filtered_fpkm)[1]="sample"

write.xlsx(filtered_fpkm, file="1为过滤值后的文件.xlsx",rowNames=F, colNames=T,quote=FALSE,sep="\t")

######使用MAD方法过滤低表达的基因#######这个过滤方法我没有用

#2025-11-22取得是前75%的基因

my_mad <- function(x){mad(x,na.rm = TRUE)} #mad时将缺失值去掉

m.mad <- apply(datExpr0,2,my_mad)

#筛选时不设置mad最小值，直接使用前75%的基因或者探针（也可以根据自身要求，更改过滤条件）。

datExpr0 <- datExpr0[,which(m.mad > quantile(m.mad, probs=seq(0, 1, 0.25))[2])]

write.xlsx(datExpr0, file="前75%mad.xlsx",rowNames=F, colNames=T,quote=FALSE,sep="\t")

#########################################样品聚类####################

sampleTree = hclust(dist(datExpr0), method = "average")

\# Plot the sample tree: Open a graphic output window of size 12 by 9 inches

\# The user should change the dimensions if the window is too large or too small.

sizeGrWindow(12,9)

#pdf(file = "1_sampleClustering.pdf", width = 12, height = 9)

par(cex = 0.6)

par(mar = c(0,4,2,0))

plot(sampleTree, main = "Sample clustering to detect outliers", sub="", xlab="", cex.lab = 1.5,

cex.axis = 1.5, cex.main = 2)

\### Plot a line to show the cut

abline(h = 4000, col = "red")##剪切高度不确定，故无红线

############### 载入性状数据## input trait data###############

#Loading clinical trait data

library(WGCNA)

######################################################input data##########################

\# The following setting is important, do not omit.

options(stringsAsFactors = FALSE)

#咱们的数据文件形式是第一行是样本组名，第二及以下行是性状名

library(openxlsx)

BB <- read.xlsx("性状数据.xlsx", startRow = 1, colNames = TRUE)

##############

\# Take a quick look at what is in the data set

dim(BB)

names(BB)

CC = as.data.frame(t(BB[,-1]))

names(CC) = BB[,1];##########如果第一行是ID命名，就写成fpkm$ID

rownames(CC) = names(BB[,-1])

allTraits =CC

dim(allTraits)

names(allTraits)

\# Form a data frame analogous to expression data that will hold the clinical traits.

fpkmSamples = rownames(datExpr0)

traitSamples =rownames(allTraits)

traitRows = match(fpkmSamples, traitSamples)

datTraits = allTraits[traitRows,]

rownames(datTraits)

collectGarbage()

\# Re-cluster samples

sampleTree2 = hclust(dist(datExpr0), method = "average")

\# Convert traits to a color representation: white means low, red means high, grey means missing entry

traitColors = numbers2colors(datTraits, signed = FALSE)

\# Plot the sample dendrogram and the colors underneath.

#sizeGrWindow(12,12)

pdf(file="2_Sample dendrogram and trait heatmap.pdf",width=12,height=12)

plotDendroAndColors(sampleTree2, traitColors,

groupLabels = names(datTraits),

main = "Sample dendrogram and trait heatmap")

dev.off()

##################################以下的文件可以直接载入并使用到后续步骤中，不需要再从头开始跑代码。

save(datExpr0, file = "样本数据处理结果.RData")

save(datTraits, file="性状处理结果.RData")

#至此，我们的性状数据和样本数据处理就完成了

##################################

write.xlsx(datExpr0, file = "数据处理结果.xlsx")

write.xlsx(datTraits,file="性状处理结果.xlsx")

#############################network constr########################################

\# Allow multi-threading within WGCNA. At present this call is necessary.

\# Any error here may be ignored but you may want to update WGCNA if you see one.

\# Caution: skip this line if you run RStudio or other third-party R environments.

\# See note above.

enableWGCNAThreads()

\# Load the data saved in the first part

#lnames = load(file = "FemaleLiver-01-dataInput.RData")

\# Choose a set of soft-thresholding powers

powers = c(1:20)

\# Call the network topology analysis function

sft = pickSoftThreshold(datExpr0, powerVector = powers, verbose = 5)

\# Plot the results:

sizeGrWindow(8, 5)

pdf(file="3_Scale independence.pdf",width=8,height=5)

par(mfrow = c(1,2))

cex1 = 0.6

\# Scale-free topology fit index as a function of the soft-thresholding power

plot(sft$fitIndices[,1], -sign(sft$fitIndices[,3])*sft$fitIndices[,2],

xlab="Soft Threshold (power)",ylab="Scale Free Topology Model Fit,signed R^2",type="n",

main = paste("Scale independence"));

text(sft$fitIndices[,1], -sign(sft$fitIndices[,3])*sft$fitIndices[,2],

labels=powers,cex=cex1,col="red");

\# this line corresponds to using an R^2 cut-off of h

abline(h=0.85,col="red")

\# Mean connectivity as a function of the soft-thresholding power

plot(sft$fitIndices[,1], sft$fitIndices[,5],

xlab="Soft Threshold (power)",ylab="Mean Connectivity", type="n",

main = paste("Mean connectivity"))

text(sft$fitIndices[,1], sft$fitIndices[,5], labels=powers, cex=cex1,col="red")

dev.off()

\# Plot the results:

sft

#GSE165321-2025-11-22此次sft是10哦

####可用sft去看使用函数估计出最佳的β值，并可运用该值到连接矩阵中IUA

######chose the softPower

softPower =sft$powerEstimate

#softPower=10

adjacency = adjacency(datExpr0, power = softPower)

##下面这一步用了我18分钟，得到了TOM矩阵

TOM=TOMsimilarity(adjacency)

dissTOM=1-TOM

save( dissTOM, file = "dissTOM.RData")

#library(WGCNA)

#library(openxlsx)

#enableWGCNAThreads()

#setwd("D:\\急性肺损伤与铁死亡数据下载及处理\\一WGCNA处理\\10.30")

\# 加载数据

#adjacency = read.xlsx("fpkm_dataInput.xlsx")

\# 将邻接矩阵转换为表达矩阵

#exprMatrix = as.matrix(adjacency)

\# 计算拓扑重叠矩阵

#该命令中的power就是之前计算得到sft给的最佳值。记得改过来哦！！

#TOM = TOMsimilarityFromExpr(exprMatrix, power = 18, verbose = 3)

\# 计算拓扑重叠矩阵的距离

#dissTOM = 1 - TOM

\# 可选：将拓扑重叠矩阵的距离转换为相似度

#simTOM <- 1 - dissTOM

\# 可选：绘制拓扑重叠矩阵的热图

#plotTOM(TOM, main = "Topological Overlap Matrix")

\# 可选：绘制拓扑重叠矩阵的距离热图

#plotDissTOM(dissTOM, main = "Dissimilarity of Topological Overlap Matrix")

\# Call the hierarchical clustering function：将基因矩阵进行聚类

geneTree = hclust(as.dist(dissTOM), method = "average");

\# Plot the resulting clustering tree (dendrogram)

#sizeGrWindow(12,9)

pdf(file="3_Gene clustering on TOM-based dissimilarity.pdf",width=12,height=9)

plot(geneTree, xlab="", sub="", main = "Gene clustering on TOM-based dissimilarity",

labels = FALSE, hang = 0.04)

dev.off()

save( geneTree,dissTOM, file = "TOManddissTOM.RData")

\# We like large modules, so we set the minimum module size relatively high:将相似性重叠好的基因分于同一模块。

##下面80数值可以更换。代表每个模块最少含有80个基因。数值越大，对应模块越少。数值越小，对应模块越多。

#2025-03-08的size是300

minModuleSize = 300

\# Module identification using dynamic tree cut:

dynamicMods = cutreeDynamic(dendro = geneTree, distM = dissTOM,

deepSplit = 2, pamRespectsDendro = FALSE,

minClusterSize = minModuleSize);

table(dynamicMods)

##举例：table(dynamicMods)结果

#0	1	2	3	4	5	6	7	8	9	10	11	12	13	14	15	16

#4206 1088 757 744 654 571 565 552 547 543 483 446 444 399 378 356 328

#17	18	19	20	21	22	23	24	25	26	27

#328 313 304 297 295 271 240 228 221 204 122

#代表一共分了27个模块，0模块对应的基因与别人没啥关系，未来会显示成灰色。各个模块内包含的基因数就是下方对应的数字

\# Convert numeric lables into colors（将模块富给不同的颜色）

dynamicColors = labels2colors(dynamicMods)

table(dynamicColors)

\# Plot the dendrogram and colors underneath

#sizeGrWindow(8,6)

pdf(file="4_Dynamic Tree Cut.pdf",width=8,height=6)

plotDendroAndColors(geneTree, dynamicColors, "Dynamic Tree Cut",

dendroLabels = FALSE, hang = 0.03,

addGuide = TRUE, guideHang = 0.05,

main = "Gene dendrogram and module colors")

dev.off()

\# Calculate eigengenes

MEList = moduleEigengenes(datExpr0, colors = dynamicColors)

MEs = MEList$eigengenes

\# Calculate dissimilarity of module eigengenes

MEDiss = 1-cor(MEs);

\# Cluster module eigengenes

METree = hclust(as.dist(MEDiss), method = "average")

\# Plot the result

#sizeGrWindow(7, 6)

pdf(file="5_Clustering of module eigengenes.pdf",width=7,height=6)

plot(METree, main = "Clustering of module eigengenes",

xlab = "", sub = "")

#下面数字0.25对应剪切高度为0.25，那么对应的相似性就是1-0.25=0.75.可以根据实际需要进行修改。改成0.2-0.3应该都可以试一试吧。数据越好，当然希望他模块的相似性越好啦。

#2025-03-08的剪切高度是0.15，及0.85相似性的模块合并

MEDissThres = 0.15

\# Plot the cut line into the dendrogram

abline(h=MEDissThres, col = "red")

dev.off()

\# Call an automatic merging function按照cutHeight = MEDissThres进行合并。

merge = mergeCloseModules(datExpr0, dynamicColors, cutHeight = MEDissThres, verbose = 3)

\# The merged module colors

mergedColors = merge$colors

\# Eigengenes of the new merged modules:

mergedMEs = merge$newMEs

\# Rename to moduleColors

moduleColors = mergedColors

\# Construct numerical labels corresponding to the colors

colorOrder = c("grey", standardColors(50))

moduleLabels = match(moduleColors, colorOrder)-1

MEs = mergedMEs

#sizeGrWindow(12, 9)

pdf(file="7_merged dynamic.pdf", width = 9, height = 6)

plotDendroAndColors(geneTree, cbind(dynamicColors, mergedColors),

c("Dynamic Tree Cut", "Merged dynamic"),

dendroLabels = FALSE, hang = 0.03,

addGuide = TRUE, guideHang = 0.05)

dev.off()

\# Save module colors and labels for use in subsequent parts将运行中产生的文件进行储存，方便以后可以直接取用，不用再从头打开一次了（此步骤用时时间也长）

save(MEs, TOM, dissTOM, moduleLabels, moduleColors, geneTree, file = "fpkm_3000-02-networkConstruction-stepByStep.RData")

#以上Rdata数据在文件夹里是R数据的储存格式。咱们千万千万不要删除fpkm_3000-02-networkConstruction-stepByStep.RData文件

#这里想再分享一下：若是做到这一步之前关闭了R语言不要着急。你把保存好的上面的R数据打开就可以啦。或者像下面一样将数据载入也行

datExpr0 <- read.xlsx("数据处理结果.xlsx", startRow = 1, colNames = TRUE)

datTraits <- read.xlsx("性状处理结果.xlsx", startRow = 1, colNames = TRUE)

##############################relate modules to external clinical triats######################################

\# Define numbers of genes and samples

nGenes = ncol(datExpr0)

nSamples = nrow(datExpr0)

moduleTraitCor = cor(MEs, datTraits, use = "p")

moduleTraitPvalue = corPvalueStudent(moduleTraitCor, nSamples)

#sizeGrWindow(10,6)

pdf(file="9_Module-trait relationships.pdf",width=14,height=10)

\# Will display correlations and their p-values

textMatrix =paste(signif(moduleTraitCor, 2), "\n(",

signif(moduleTraitPvalue, 1), ")", sep = "")

dim(textMatrix) = dim(moduleTraitCor)

par(mar = c(6, 8.5, 3, 3))

\# Display the correlation values within a heatmap plot

labeledHeatmap(Matrix = moduleTraitCor,

xLabels = names(datTraits),

yLabels = names(MEs),

ySymbols = names(MEs),

colorLabels = FALSE,

colors = greenWhiteRed(50),

textMatrix = textMatrix,

setStdMargins = FALSE,

cex.text = 0.5,

zlim = c(-1,1),

main = paste("Module-trait relationships"))

dev.off()

######## Define variable weight containing all column of datTraits

###MM and GS

\# names (colors) of the modules

modNames = substring(names(MEs), 3)

geneModuleMembership = as.data.frame(cor(datExpr0, MEs, use = "p"))

MMPvalue = as.data.frame(corPvalueStudent(as.matrix(geneModuleMembership), nSamples))

names(geneModuleMembership) = paste("MM", modNames, sep="")

names(MMPvalue) = paste("p.MM", modNames, sep="")

#names of those trait

traitNames=names(datTraits)

geneTraitSignificance = as.data.frame(cor(datExpr0, datTraits, use = "p"))

GSPvalue = as.data.frame(corPvalueStudent(as.matrix(geneTraitSignificance), nSamples))

names(geneTraitSignificance) = paste("GS.", traitNames, sep="")

names(GSPvalue) = paste("p.GS.", traitNames, sep="")

##########example:royalblue and CK

module="skyblue"

column = match(module, modNames)

moduleGenes = moduleColors==module

trait="RIF"

traitColumn=match(trait,traitNames)

sizeGrWindow(7, 7)

par(mfrow = c(1,1))

verboseScatterplot(abs(geneModuleMembership[moduleGenes, column]),

abs(geneTraitSignificance[moduleGenes, traitColumn]),

xlab = paste("Module Membership in", module, "module"),

ylab = paste("Gene significance for ",trait),

main = paste("Module membership vs. gene significance\n"),

cex.main = 1.2, cex.lab = 1.2, cex.axis = 1.2, col = module)

######下面循环把所有的颜色模块和性状都循环出来啦。可以用下面的写出来在找哈。

for (trait in traitNames){

traitColumn=match(trait,traitNames)

for (module in modNames){

column = match(module, modNames)

moduleGenes = moduleColors==module

if (nrow(geneModuleMembership[moduleGenes,]) > 1){####进行这部分计算必须每个模块内基因数量大于2，由于前面设置了最小数量是30，这里可以不做这个判断，但是grey有可能会出现1个gene,它会导致代码运行的时候中断，故设置这一步

#sizeGrWindow(7, 7)

pdf(file=paste("9_", trait, "_", module,"_Module membership vs gene significance.pdf",sep=""),width=7,height=7)

par(mfrow = c(1,1))

verboseScatterplot(abs(geneModuleMembership[moduleGenes, column]),

abs(geneTraitSignificance[moduleGenes, traitColumn]),

xlab = paste("Module Membership in", module, "module"),

ylab = paste("Gene significance for ",trait),

main = paste("Module membership vs. gene significance\n"),

cex.main = 1.2, cex.lab = 1.2, cex.axis = 1.2, col = module)

dev.off()

}

}

}

\#####

names(datExpr0)

probes = names(datExpr0)

#################export GS and MM###############

geneInfo0 = data.frame(probes= probes,

moduleColor = moduleColors)

for (Tra in 1:ncol(geneTraitSignificance))

{

oldNames = names(geneInfo0)

geneInfo0 = data.frame(geneInfo0, geneTraitSignificance[,Tra],

GSPvalue[, Tra])

names(geneInfo0) = c(oldNames,names(geneTraitSignificance)[Tra],

names(GSPvalue)[Tra])

}

for (mod in 1:ncol(geneModuleMembership))

{

oldNames = names(geneInfo0)

geneInfo0 = data.frame(geneInfo0, geneModuleMembership[,mod],

MMPvalue[, mod])

names(geneInfo0) = c(oldNames,names(geneModuleMembership)[mod],

names(MMPvalue)[mod])

}

geneOrder =order(geneInfo0$moduleColor)

geneInfo = geneInfo0[geneOrder, ]

write.table(geneInfo, file = "10_GS_and_MM.xls",sep="\t",row.names=F)

####################################################Visualizing the gene network对基因与基因相关性进行可视化#######################################################

nGenes = ncol(datExpr0)

nSamples = nrow(datExpr0)

#一：以下内容是画所有基因及模块图，此步骤所需时间长

\# Transform dissTOM with a power to make moderately strong connections more visible in the heatmap

plotTOM = dissTOM^7

\# Set diagonal to NA for a nicer plot

diag(plotTOM) = NA

\# Call the plot function

#sizeGrWindow(9,9)本步骤用时较长

pdf(file="8_Network heatmap plot_all gene.pdf",width=9, height=9)

TOMplot(plotTOM, geneTree, moduleColors, main = "Network heatmap plot, all genes")

dev.off()

#二：以下内容选定画3000个基因及图片

nSelect = 3000

\# For reproducibility, we set the random seed

set.seed(10)

select = sample(nGenes, size = nSelect)

selectTOM = dissTOM[select, select]

\# There's no simple way of restricting a clustering tree to a subset of genes, so we must re-cluster.

selectTree = hclust(as.dist(selectTOM), method = "average")

selectColors = moduleColors[select]

\# Open a graphical window

#sizeGrWindow(9,9)

\# Taking the dissimilarity to a power, say 10, makes the plot more informative by effectively changing

\# the color palette; setting the diagonal to NA also improves the clarity of the plot

plotDiss = selectTOM^7

diag(plotDiss) = NA

pdf(file="13_Network heatmap plot_selected genes.pdf",width=9, height=9)

TOMplot(plotDiss, selectTree, selectColors, main = "Network heatmap plot, selected genes")

dev.off()

####################################################Visualizing the gene network of eigengenes对基因模块进行可视化####################################################

#sizeGrWindow(5,7.5)

pdf(file="10_Eigengene dendrogram and Eigengene adjacency heatmap.pdf", width=5, height=7.5)

par(cex = 0.9)

plotEigengeneNetworks(MEs, "", marDendro = c(0,4,1,2), marHeatmap = c(3,4,1,2), cex.lab = 0.8, xLabelsAngle= 90)

dev.off()

#or devide into two parts

\# Plot the dendrogram

#sizeGrWindow(6,6);

pdf(file="11_Eigengene dendrogram_2.pdf",width=6, height=6)

par(cex = 1.0)

plotEigengeneNetworks(MEs, "Eigengene dendrogram", marDendro = c(0,4,2,0), plotHeatmaps = FALSE)

dev.off()

pdf(file="12_Eigengene adjacency heatmap_2.pdf",width=6, height=6)

\# Plot the heatmap matrix (note: this plot will overwrite the dendrogram plot)

par(cex = 1.0)

plotEigengeneNetworks(MEs, "Eigengene adjacency heatmap", marHeatmap = c(3,4,2,2), plotDendrograms = FALSE, xLabelsAngle = 90)

dev.off()

###########################Exporting to Cytoscape all one by one ##########################

\# Select each module

for (mod in 1:nrow(table(moduleColors)))

{

modules = names(table(moduleColors))[mod]

\# Select module probes

probes = names(datExpr0)

inModule = (moduleColors == modules)

modProbes = probes[inModule]

modGenes = modProbes

\# Select the corresponding Topological Overlap

modTOM = TOM[inModule, inModule]

dimnames(modTOM) = list(modProbes, modProbes)

\# Export the network into edge and node list files Cytoscape can read

cyt = exportNetworkToCytoscape(modTOM,

edgeFile = paste("CytoscapeInput-edges-", modules , ".txt", sep=""),

nodeFile = paste("CytoscapeInput-nodes-", modules, ".txt", sep=""),

weighted = TRUE,

threshold = 0.02,

nodeNames = modProbes,

altNodeNames = modGenes,

nodeAttr = moduleColors[inModule])

}

\# 计算模块特征基因

MEs = moduleEigengenes(datExpr0, moduleColors)$eigengenes

\# 输出每个模块的特征基因列表

for (i in seq_len(ncol(MEs))) {

print(paste("Module", i, ":"))

genes = rownames(subset(datExpr0, select = colnames(datExpr0)[which(moduleColors == i)]))

print(genes)

}

\# 或者按照模块颜色输出

for (modCol in unique(moduleColors)) {

print(paste("Module", modCol, ":"))

genes = rownames(subset(datExpr0, select = colnames(datExpr0)[which(moduleColors == modCol)]))

print(genes)

}

library(limma)

library(org.Hs.eg.db)

library(clusterProfiler)

library(enrichplot)

gmtFile="c2.cp.kegg.v7.4.symbols.gmt"

setwd("D:\\IUA-从头到尾\\GSE165321LIMMA+GO+KEGG+GSEA")

rt=read.table("diffExp.txt", header=T, sep="\t", check.names=F)

logFC=as.vector(rt[,2])

names(logFC)=as.vector(rt[,1])

logFC=sort(logFC, decreasing=T)

gmt=read.gmt(gmtFile)

kk=GSEA(logFC, TERM2GENE=gmt, pvalueCutoff = 1)

kkTab=as.data.frame(kk)

kkTab=kkTab[kkTab$p.adjust<0.05,]

write.table(kkTab,file="165321All-LIMMA.result.txt",sep="\t",quote=F,row.names = F)

termNum=6

kkUp=kkTab[kkTab$NES>0,]

if(nrow(kkUp)>=termNum){

showTerm=row.names(kkUp)[1:termNum]

gseaplot=gseaplot2(kk, showTerm, base_size=8, title="Enriched in IUA")

pdf(file="165321All-LIMMA.pdf", width=7, height=5.5)

print(gseaplot)

dev.off()

}

termNum=6

kkUp=kkTab[kkTab$NES>0,]

if(nrow(kkUp)>=termNum){

showTerm=row.names(kkUp)[1:termNum]

\# 创建更美观的显示名称

pretty_names <- tolower(gsub("KEGG_", "", showTerm))

term_mapping <- setNames(pretty_names, showTerm)

\# 创建kk对象的副本

kk_plot <- kk

\# 修改显示名称

for(term in showTerm){

kk_plot@result[term, "Description"] <- term_mapping[term]

}

gseaplot=gseaplot2(kk_plot, showTerm, base_size=10, title="Enriched in IUA")

pdf(file="2-165321All-LIMMA.pdf", width=7, height=5.5)

print(gseaplot)

dev.off()

}

library("clusterProfiler")

library("org.Hs.eg.db")

library("enrichplot")

library("ggplot2")

pvalueFilter=0.05

qvalueFilter=0.05

colorSel="qvalue"

if(qvalueFilter>0.05){

colorSel="pvalue"

}

setwd("D:\\IUA-从头到尾\\GSE165321LIMMA+GO+KEGG+GSEA")

rt=read.table("165321-mRNA-diff.txt", header=T, sep="\t", check.names=F)

genes=as.vector(rt[,1])

entrezIDs=mget(genes, org.Hs.egSYMBOL2EG, ifnotfound=NA)

entrezIDs=as.character(entrezIDs)

rt=cbind(rt,entrezID=entrezIDs)

gene=entrezIDs[entrezIDs!="NA"]

\# 设置KEGG镜像源（如果可用）

library(clusterProfiler)

library(KEGGREST)

\# 尝试设置代理（如果需要）

\# Sys.setenv(http_proxy="http://your_proxy:port")

\# Sys.setenv(https_proxy="https://your_proxy:port")

\# 或者尝试使用其他网络设置

options(timeout = 400) # 增加超时时间

\# 重新尝试

kk <- enrichKEGG(gene=gene, organism="hsa", pvalueCutoff=1, qvalueCutoff=1)

#kk=enrichKEGG(gene=gene, organism="hsa", pvalueCutoff=1, qvalueCutoff=1)

KEGG=as.data.frame(kk)

KEGG$geneID=as.character(sapply(KEGG$geneID,function(x)paste(rt$id[match(strsplit(x,"/")[[1]],as.character(rt$entrezID))],collapse="/")))

KEGG=KEGG[(KEGG$pvalue<pvalueFilter & KEGG$qvalue<qvalueFilter),]

#???渻??????

write.table(KEGG, file="2KEGG.txt", sep="\t", quote=F, row.names = F)

showNum=10

if(nrow(KEGG)<showNum){

showNum=nrow(KEGG)

}

pdf(file="KEGG柱状图.pdf", width=8, height=7)

barplot(kk, drop=TRUE, showCategory=showNum, color=colorSel)

dev.off()

pdf(file="KEGG气泡图.pdf", width = 8, height = 7)

dotplot(kk, showCategory=showNum, orderBy="GeneRatio", color=colorSel)

dev.off()

setwd("D:\\IUA-从头到尾\\GSE215968-基质细胞-IUA-CON-TAGLV-TNS1小提琴图")

#开启半自动注释阶段。

#生成IUA组分类和标记，以便后续提取目标群体的基因表达量

seuratdta<-readRDS(file = "IUA-UMAP降维后的结果.rds")

hpca.se<-readRDS(file = "IUA-hpca.se.rds")

countdt<-seuratdta@assays$RNA$counts

clusterdt<-seuratdta@meta.data$seurat_clusters

\# 进性分类细胞群

seuratdta_label <- SingleR(test = countdt,

ref = hpca.se,

labels = hpca.se$label.main,

clusters = clusterdt)

\# 重命名聚类

new.cluster.ids <- seuratdta_label$pruned.labels

names(new.cluster.ids) <- levels(seuratdta)

seuratdta <- RenameIdents(seuratdta, new.cluster.ids)

print(seuratdta_label)

\# 提取聚类编号与细胞类型的对应关系

cluster_celltype_mapping <- data.frame(

Cluster = rownames(seuratdta_label),

CellType = seuratdta_label$pruned.labels

)

)

\# 将细胞类型添加到seurat对象的metadata中

seuratdta$celltype <- celltype_mapping_iua$CellType[match(seuratdta$seurat_clusters, celltype_mapping_iua$Cluster)]

\# 设置细胞类型为标识

Idents(seuratdta) <- "celltype"

\# 创建自定义颜色方案

celltypes <- unique(na.omit(celltype_mapping_iua$CellType))

n_celltypes <- length(celltypes)

\# 生成足够的颜色

if (n_celltypes <= 8) {

colors <- brewer.pal(n_celltypes, "Set2")

} else if (n_celltypes <= 12) {

colors <- brewer.pal(n_celltypes, "Set3")

} else {

colors <- colorRampPalette(brewer.pal(8, "Set2"))(n_celltypes)

}

\# 为每个细胞类型分配颜色

names(colors) <- celltypes

\# 计算每个细胞类型的中心点用于标签定位

umap_coords <- as.data.frame(seuratdta@reductions$umap@cell.embeddings)

colnames(umap_coords) <- c("UMAP_1", "UMAP_2")

umap_coords$celltype <- seuratdta$celltype

celltype_centers <- umap_coords %>%

group_by(celltype) %>%

summarise(

UMAP_1 = median(UMAP_1),

UMAP_2 = median(UMAP_2)

)

seurat_healthy <- readRDS(file = "CON-UMAP降维后的结果.rds") # 假设这是健康组数据

\# 使用SingleR进行细胞注释（如果尚未注释）

\# 将参考数据保存为健康组特定的变量名

hpca_se_healthy <- readRDS(file = "CON-hpca.se.rds")

countdt_healthy <- seurat_healthy@assays$RNA$counts

clusterdt_healthy <- seurat_healthy@meta.data$seurat_clusters

\# 进行细胞分类

seurat_healthy_label <- SingleR(test = countdt_healthy,

ref = hpca_se_healthy,

labels = hpca_se_healthy$label.main,

clusters = clusterdt_healthy)

\# 查看注释结果

print(seurat_healthy_label)

\# 提取聚类编号与细胞类型的对应关系

cluster_celltype_mapping_healthy <- data.frame(

Cluster = rownames(seurat_healthy_label),

CellType = seurat_healthy_label$pruned.labels

)

\# 打印所有聚类的细胞类型

print(cluster_celltype_mapping_healthy)

\# 将细胞类型添加到健康组seurat对象的metadata中

seurat_healthy$celltype <- celltype_mapping_healthy$CellType[match(seurat_healthy$seurat_clusters, celltype_mapping_healthy$Cluster)]

\# 设置细胞类型为标识

Idents(seurat_healthy) <- "celltype"

\# 创建自定义颜色方案

celltypes_healthy <- unique(na.omit(celltype_mapping_healthy$CellType))

n_celltypes_healthy <- length(celltypes_healthy)

\# 生成足够的颜色

if (n_celltypes_healthy <= 8) {

colors_healthy <- brewer.pal(n_celltypes_healthy, "Set2")

} else if (n_celltypes_healthy <= 12) {

colors_healthy <- brewer.pal(n_celltypes_healthy, "Set3")

} else {

colors_healthy <- colorRampPalette(brewer.pal(8, "Set2"))(n_celltypes_healthy)

}

\# 为每个细胞类型分配颜色

names(colors_healthy) <- celltypes_healthy

\# 计算每个细胞类型的中心点用于标签定位

umap_coords_healthy <- as.data.frame(seurat_healthy@reductions$umap@cell.embeddings)

colnames(umap_coords_healthy) <- c("UMAP_1", "UMAP_2")

umap_coords_healthy$celltype <- seurat_healthy$celltype

celltype_centers_healthy <- umap_coords_healthy %>%

group_by(celltype) %>%

summarise(

UMAP_1 = median(UMAP_1),

UMAP_2 = median(UMAP_2)

)

\# 添加细胞类型注释 (这里已经添加过了，可以删除重复的部分)

\# seuratdta$celltype <- celltype_mapping_iua$CellType[match(seuratdta$seurat_clusters, celltype_mapping_iua$Cluster)]

\# seurat_healthy$celltype <- celltype_mapping_healthy$CellType[match(seurat_healthy$seurat_clusters, celltype_mapping_healthy$Cluster)]

\# 设置分组信息

seuratdta$group <- "IUA"

seurat_healthy$group <- "Healthy"

\# 提取子宫内膜基质细胞

stromal_iua <- subset(seuratdta, subset = celltype == "Stromal cells")

stromal_healthy <- subset(seurat_healthy, subset = celltype == "Stromal cells")

\# 打印细胞数量信息

cat("细胞数量统计:\n")

cat("IUA组基质细胞数:", ncol(stromal_iua), "\n")

cat("健康组基质细胞数:", ncol(stromal_healthy), "\n")

cat("IUA组基质细胞占比:", ncol(stromal_iua)/ncol(seuratdta)*100, "%\n")

cat("健康组基质细胞占比:", ncol(stromal_healthy)/ncol(seurat_healthy)*100, "%\n")

\# 合并数据

stromal_combined <- merge(stromal_healthy, y = stromal_iua, add.cell.ids = c("Healthy", "IUA"))

\# 提取表达数据 - 将AZIN1/ODC1替换为TAGLN/TNS1

expr_data <- FetchData(stromal_combined, vars = c("TAGLN", "TNS1", "group"))

\# 分析零表达比例 - 将AZIN1/ODC1替换为TAGLN/TNS1

zero_expr_stats <- expr_data %>%

group_by(group) %>%

summarise(

TAGLN_zero = sum(TAGLN == 0) / n(),

TNS1_zero = sum(TNS1 == 0) / n(),

n_cells = n()

)

print(zero_expr_stats)

\# 创建非零表达细胞数据

create_nonzero_cell_data <- function(expr_data, gene) {

nonzero_data <- expr_data[expr_data[[gene]] > 0, ]

return(nonzero_data)

}

\# 为TAGLN和TNS1创建非零表达细胞数据 - 替换变量名

nonzero_TAGLN_data <- create_nonzero_cell_data(expr_data, "TAGLN")

nonzero_TNS1_data <- create_nonzero_cell_data(expr_data, "TNS1")

\# 修正后的非零表达细胞小提琴图函数

create_nonzero_violin_plot <- function(nonzero_data, gene, y_limits = NULL) {

\# 计算每组的细胞总数（用于计算百分比）

total_cells_per_group <- expr_data %>%

group_by(group) %>%

summarise(total = n())

\# 计算每组的统计量

stats <- nonzero_data %>%

group_by(group) %>%

summarise(

mean_expr = mean(.data[[gene]]),

n_cells = n()

) %>%

left_join(total_cells_per_group, by = "group") %>%

mutate(percentage_total = n_cells / total * 100)

\# 使用红色系和蓝色系配色

fill_colors <- c("Healthy" = "#F8766D", "IUA" = "#619CFF")

\# 创建图

p <- ggplot(nonzero_data, aes(x = group, y = .data[[gene]], fill = group)) +

geom_violin(scale = "width", trim = TRUE, alpha = 0.8, color = NA) +

geom_boxplot(width = 0.15, fill = "white", alpha = 0.7, outlier.size = 0.3,

outlier.color = "gray40", outlier.alpha = 0.5) +

\# 添加均值点

geom_point(data = stats, aes(x = group, y = mean_expr),

color = "black", size = 3, shape = 18) +

\# 添加均值文本标签

geom_text(data = stats, aes(x = group, y = mean_expr,

label = sprintf("Mean: %.3f", mean_expr)),

vjust = -1.2, color = "black", size = 3.2, fontface = "bold") +

\# 添加细胞数量标签

geom_text(data = stats, aes(x = group, y = min(nonzero_data[[gene]], na.rm = TRUE),

label = sprintf("n=%d (%.1f%%)", n_cells, percentage_total)),

vjust = 1.5, color = "black", size = 3) +

\# 添加统计检验

stat_compare_means(

method = "wilcox.test",

comparisons = list(c("Healthy", "IUA")),

label = "p.format",

label.y = max(nonzero_data[[gene]], na.rm = TRUE) * 1.15,

size = 4

) +

scale_fill_manual(values = fill_colors) +

labs(

title = gene,

x = "Group",

y = "Expression Level"

) +

theme_classic() +

theme(

plot.title = element_text(hjust = 0.5, face = "bold", size = 14),

legend.position = "none",

axis.text.x = element_text(angle = 0, hjust = 0.5, size = 11)

)

\# 如果指定了y轴范围，则设置

if (!is.null(y_limits)) {

p <- p + ylim(y_limits)

}

return(p)

}

\# 创建两个基因的非零表达图 - 替换变量名

p_TAGLN_nonzero <- create_nonzero_violin_plot(nonzero_TAGLN_data, "TAGLN")

p_TNS1_nonzero <- create_nonzero_violin_plot(nonzero_TNS1_data, "TNS1")

\# 创建组合图 - 并排显示 - 修正变量名

combined_plot <- p_TAGLN_nonzero + p_TNS1_nonzero +

plot_annotation(

title = "TAGLN and TNS1 Expression in Non-zero Stromal Cells",

theme = theme(plot.title = element_text(hjust = 0.5, face = "bold", size = 16))

)

\# 保存组合图

pdf("Nonzero_Cells_TAGLN_TNS1_SideBySide.pdf", 12, 6)

print(combined_plot)

dev.off()

\# 打印非零细胞的统计摘要 - 修正基因名和变量名

cat("=== 非零表达细胞统计摘要 ===\n")

for(gene in c("TAGLN", "TNS1")) {

if(gene == "TAGLN") {

nonzero_data <- nonzero_TAGLN_data

} else {

nonzero_data <- nonzero_TNS1_data

}

healthy_expr <- nonzero_data[nonzero_data$group == "Healthy", gene]

iua_expr <- nonzero_data[nonzero_data$group == "IUA", gene]

test_result <- wilcox.test(healthy_expr, iua_expr)

cat(sprintf("\n%s (非零表达细胞):\n", gene))

cat(sprintf(" 健康组 - 细胞数: %d, 均值: %.3f\n",

length(healthy_expr), mean(healthy_expr)))

cat(sprintf(" IUA组 - 细胞数: %d, 均值: %.3f\n",

length(iua_expr), mean(iua_expr)))

cat(sprintf(" Wilcoxon检验 p值: %.4f\n", test_result$p.value))

}

cat("\n=== 图表生成完成 ===\n")

cat("已生成以下文件:\n")

cat("1. Nonzero_Cells_TAGLN_TNS1_SideBySide.pdf - 并排显示的非零细胞表达图\n")

setwd("D:\\IUA-从头到尾\\GSE215968-单细胞拟时续-IUA组")

\# 读取降维后的UMAP结果

seuratdta <- readRDS(file = "2UMAP降维后的结果.rds")

hpca.se <- readRDS(file = "2hpca.se.rds")

countdt <- seuratdta@assays$RNA$counts

clusterdt <- seuratdta@meta.data$seurat_clusters

\# 进行细胞群分类

seuratdta_label <- SingleR(test = countdt,

ref = hpca.se,

labels = hpca.se$label.main,

clusters = clusterdt)

\# 重命名聚类

new.cluster.ids <- seuratdta_label$pruned.labels

names(new.cluster.ids) <- levels(seuratdta)

seuratdta <- RenameIdents(seuratdta, new.cluster.ids)

\# 生成UMAP图

umapplot2 <- UMAPPlot(seuratdta, label = TRUE, pt.size = 1.5)

\# 查看SingleR的注释结果

print(seuratdta_label)

\# ==================== 检查可用的细胞类型 ====================

cat("当前Seurat对象中的细胞类型：\n")

print(levels(seuratdta))

print(unique(seuratdta@active.ident))

cat("\n细胞类型分布：\n")

print(table(seuratdta@active.ident))

\# 让用户选择细胞类型或自动选择

all_cell_types <- unique(seuratdta@active.ident)

\# 查找包含"stem"的细胞类型（不区分大小写）

stem_cell_types <- grep("stem|Stem", all_cell_types, value = TRUE, ignore.case = TRUE)

if (length(stem_cell_types) > 0) {

target_cell_type <- stem_cell_types[1]

cat("找到干细胞相关细胞类型，使用：", target_cell_type, "\n")

} else {

\# 如果没有找到干细胞类型，使用第一个细胞类型

target_cell_type <- all_cell_types[1]

cat("未找到干细胞类型，使用：", target_cell_type, "\n")

}

\# ==================== 数据预处理：提取目标细胞类型和基因 ====================

\# 选择高变基因前1000个，修改您要敲除的基因

ngene <- 1000

target_gene <- "TAGLN" # 修改为TAGLN

\# 合并高变基因和目标基因（去重）

features_keep <- unique(c(Seurat::VariableFeatures(seuratdta)[1:ngene], target_gene))

\# 提取目标细胞类型

subrds <- subset(seuratdta,

features = features_keep, # 高变基因子集

idents = target_cell_type) # 使用正确的细胞类型

\# 查看子集的细胞类型分布

cat("\n子集细胞类型分布：\n")

print(table(subrds@active.ident))

\# 检查子集大小

cat("\n子集大小：\n")

cat("细胞数：", ncol(subrds), "\n")

cat("基因数：", nrow(subrds), "\n")

\# 可选：降采样细胞（如果细胞数太多）

ncell <- 500

if (ncol(subrds) > ncell) {

cat("\n细胞数太多（", ncol(subrds), "），进行降采样至", ncell, "个细胞\n")

subrds <- subset(subrds, downsample = ncell)

} else {

cat("\n细胞数：", ncol(subrds), "，无需降采样\n")

}

\# ==================== 提取表达矩阵 ====================

mat <- Seurat::GetAssayData(object = subrds,

slot = "counts",

assay = "RNA")

\# 转换为数据框（scTenifoldKnk兼容输入格式）

mat <- as.data.frame(mat)

\# 查看矩阵维度

cat("\n表达矩阵维度：\n")

print(dim(mat))

\# ==================== 运行scTenifoldKnk分析 ====================

knk_gene <- "TAGLN" # 修改为TAGLN

\# 检查敲除基因是否存在于矩阵中

if (!knk_gene %in% rownames(mat)) {

stop(paste("敲除基因", knk_gene, "不在表达矩阵中，请检查基因名！"))

} else {

cat("目标基因", knk_gene, "在表达矩阵中\n")

}

\# 运行scTenifoldKnk核心函数

knk_res <- scTenifoldKnk(

countMatrix = mat, # 输入表达矩阵

gKO = knk_gene, # 目标敲除基因

qc_mtThreshold = 0.1, # 线粒体基因占比阈值

qc_minLSize = 1000, # 细胞最小基因数阈值

nc_lambda = 0, # 邻接矩阵方向性权重

nc_nNet = 10, # 生成的随机网络数量

nc_nCells = 500, # 每个随机网络的下采样细胞数

nc_nComp = 3, # PCA降维的主成分数

nc_scaleScores = TRUE, # 对网络得分进行中心化和标准化

nc_symmetric = FALSE, # 邻接矩阵非对称

nc_q = 0.9, # 保留网络关联性前90%的边

td_K = 3, # CP张量分解的一阶张量数量

td_maxIter = 1000, # 张量分解最大迭代次数

td_maxError = 1e-05, # 张量分解误差容忍度

td_nDecimal = 3, # 结果保留3位小数

ma_nDim = 2	# 流形对齐后的低维特征空间维度

)

\# ==================== 提取差异调控基因 ====================

knk_df <- knk_res$diffRegulation %>%

\# 计算log2倍变化

dplyr::mutate(logFC = log2(knk_res$diffRegulation$FC)) %>%

\# 过滤掉敲除基因本身

dplyr::filter(gene != knk_gene) %>%

\# 确保数值列是数值类型

mutate(across(c(distance, Z, FC, p.value, p.adj, logFC), as.numeric))

\# ==================== 筛选显著基因（p.value < 0.05） ====================

\# 定义筛选阈值

p_value_threshold <- 0.05

logFC_threshold <- 0.5 # 可根据需要调整

\# 策略1：先筛选p.value < 0.05的基因

significant_genes <- knk_df %>%

filter(p.value < p_value_threshold) %>%

arrange(p.value)

\# 检查筛选结果

cat("\n筛选统计：\n")

cat("总基因数:", nrow(knk_df), "\n")

cat("p.value <", p_value_threshold, "的基因数:", nrow(significant_genes), "\n")

\# 如果显著基因太多，再按logFC绝对值筛选

if (nrow(significant_genes) > 1000) {

significant_genes <- significant_genes %>%

filter(abs(logFC) > logFC_threshold) %>%

slice_head(n = 1000)

cat("进一步筛选：|logFC| >", logFC_threshold, "的基因数:", nrow(significant_genes), "\n")

}

\# 如果筛选后基因数为0，放宽条件

if (nrow(significant_genes) == 0) {

cat("警告：没有满足p.value <", p_value_threshold, "的基因，将使用logFC绝对值最大的前100个基因\n")

significant_genes <- knk_df %>%

arrange(-abs(logFC)) %>%

slice_head(n = 100)

}

\# 使用筛选后的基因

knk_df_filtered <- significant_genes

cat("最终用于可视化的基因数:", nrow(knk_df_filtered), "\n")

\# ==================== 保存结果 ====================

\# 保存为CSV文件

write.csv(knk_df_filtered,

file = paste0(knk_gene, "_significant_genes_p0.05.csv"),

row.names = FALSE,

na = "")

\# 使用readr保存

write_csv(knk_df_filtered,

path = paste0(knk_gene, "_significant_genes_p0.05_readr.csv"))

\# ==================== 定义关键基因集 ====================

\# 定义胶原相关基因

collagen_genes <- c("COL1A1", "COL1A2", "COL2A1", "COL3A1", "COL4A1", "COL4A2",

"COL5A1", "COL5A2", "COL6A1", "COL6A2", "COL6A3",

"COL7A1", "COL8A1", "COL9A1", "COL10A1", "COL11A1", "COL12A1")

\# 定义肌成纤维细胞相关基因（包含TAGLN）

myofibroblast_genes <- c("ACTA2", "TAGLN", "TNS1", "MYH11", "MYLK", "MYL9", "DES",

"VIM", "FN1", "CTGF", "POSTN", "TNC", "SPARC")

\# 定义纤维化相关基因

fibrosis_genes <- c("TGFB1", "TGFB2", "TGFB3", "SMAD2", "SMAD3", "SMAD4",

"MMP2", "MMP9", "TIMP1", "TIMP2", "IL6", "IL1B", "TNF",

"PDGFB", "VEGFA", "FGF2")

\# 合并所有需要突出显示的基因

highlight_genes <- unique(c(collagen_genes, myofibroblast_genes, fibrosis_genes))

\# 检查哪些基因在筛选后的数据中

genes_in_data <- highlight_genes[highlight_genes %in% knk_df_filtered$gene]

cat("\n在数据中找到的关键基因：\n")

print(genes_in_data)

cat("\n总数：", length(genes_in_data), "\n")

\# ==================== 准备绘图数据 ====================

\# 设置最大显示基因数

max_display <- 30

\# 分割数据为上调和下调基因

up_genes <- knk_df_filtered %>%

filter(logFC > 0) %>%

arrange(-logFC) %>%

slice_head(n = min(max_display, nrow(.)))

down_genes <- knk_df_filtered %>%

filter(logFC < 0) %>%

arrange(logFC) %>%

slice_head(n = min(max_display, nrow(.)))

cat("\n上调基因数:", nrow(up_genes), "\n")

cat("下调基因数:", nrow(down_genes), "\n")

\# 添加基因类型列和显著性标记

if (nrow(up_genes) > 0) {

up_genes <- up_genes %>%

mutate(

gene_type = ifelse(gene %in% highlight_genes, "Highlighted", "Normal"),

gene_type = factor(gene_type, levels = c("Normal", "Highlighted")),

significance = case_when(

p.value < 0.001 ~ "***",

p.value < 0.01 ~ "**",

p.value < 0.05 ~ "*",

TRUE ~ ""

)

)

}

if (nrow(down_genes) > 0) {

down_genes <- down_genes %>%

mutate(

gene_type = ifelse(gene %in% highlight_genes, "Highlighted", "Normal"),

gene_type = factor(gene_type, levels = c("Normal", "Highlighted")),

significance = case_when(

p.value < 0.001 ~ "***",

p.value < 0.01 ~ "**",

p.value < 0.05 ~ "*",

TRUE ~ ""

)

)

}

\# ==================== 创建上调基因条形图 ====================

if (nrow(up_genes) > 0) {

p_up <- ggplot(up_genes, aes(x = reorder(gene, logFC), y = logFC)) +

geom_bar(stat = "identity",

aes(fill = gene_type),

color = "gray30",

linewidth = 0.3) +

scale_fill_manual(values = c("Normal" = "pink", "Highlighted" = "pink"),

labels = c("Normal" = "Other Genes", "Highlighted" = "Key Genes"),

name = "Gene Type") +

coord_flip() +

labs(title = paste("Top", nrow(up_genes), "Up-regulated Genes after", knk_gene, "Knockout"),

x = "Gene",

y = "log2(Fold Change)") +

theme_minimal() +

theme(

axis.text.y = element_text(size = 10, face = "plain"),

axis.text.x = element_text(size = 10),

axis.title = element_text(size = 12, face = "bold"),

plot.title = element_text(size = 14, face = "bold", hjust = 0.5, margin = margin(b = 10)),

legend.position = "bottom",

legend.title = element_text(face = "bold"),

panel.grid.major.y = element_blank(),

panel.grid.minor = element_blank()

) +

\# 为突出显示的基因添加标签

geom_text(data = subset(up_genes, gene_type == "Highlighted"),

aes(label = gene, y = 0.1),

hjust = 0, color = "darkred", size = 3, fontface = "bold") +

\# 添加显著性标记

geom_text(aes(label = significance),

vjust = 0.5, hjust = -0.2,

color = "black", size = 4)

} else {

p_up <- NULL

cat("没有显著上调基因\n")

}

\# ==================== 创建下调基因条形图 ====================

if (nrow(down_genes) > 0) {

p_down <- ggplot(down_genes, aes(x = reorder(gene, -logFC), y = logFC)) +

geom_bar(stat = "identity",

aes(fill = gene_type),

color = "gray30",

linewidth = 0.3) +

scale_fill_manual(values = c("Normal" = "pink", "Highlighted" = "#4ECDC4"),

labels = c("Normal" = "Other Genes", "Highlighted" = "Key Genes"),

name = "Gene Type") +

coord_flip() +

labs(title = paste("Top", nrow(down_genes), "Down-regulated Genes after", knk_gene, "Knockout"),

x = "Gene",

y = "log2(Fold Change)") +

theme_minimal() +

theme(

axis.text.y = element_text(size = 10, face = "plain"),

axis.text.x = element_text(size = 10),

axis.title = element_text(size = 12, face = "bold"),

plot.title = element_text(size = 14, face = "bold", hjust = 0.5, margin = margin(b = 10)),

legend.position = "bottom",

legend.title = element_text(face = "bold"),

panel.grid.major.y = element_blank(),

panel.grid.minor = element_blank()

) +

\# 为突出显示的基因添加标签

geom_text(data = subset(down_genes, gene_type == "Highlighted"),

aes(label = gene, y = -0.1),

hjust = 1, color = "darkblue", size = 3, fontface = "bold") +

\# 添加显著性标记

geom_text(aes(label = significance),

vjust = 0.5, hjust = 1.2,

color = "black", size = 4)

} else {

p_down <- NULL

cat("没有显著下调基因\n")

}

\# ==================== 组合图表 ====================

if (!is.null(p_up) && !is.null(p_down)) {

combined_plot <- p_up / p_down +

plot_layout(guides = "collect") &

theme(legend.position = "bottom")

} else if (!is.null(p_up)) {

combined_plot <- p_up

} else if (!is.null(p_down)) {

combined_plot <- p_down

} else {

\# 如果没有显著基因，创建所有基因的图（只显示前50个）

cat("没有显著差异表达基因，创建所有基因的图\n")

all_genes <- knk_df %>%

arrange(-abs(logFC)) %>%

slice_head(n = 50) %>%

mutate(

gene_type = ifelse(gene %in% highlight_genes, "Highlighted", "Normal"),

gene_type = factor(gene_type, levels = c("Normal", "Highlighted")),

significance = case_when(

p.value < 0.001 ~ "***",

p.value < 0.01 ~ "**",

p.value < 0.05 ~ "*",

TRUE ~ ""

),

direction = ifelse(logFC > 0, "Up", "Down")

)

combined_plot <- ggplot(all_genes, aes(x = reorder(gene, logFC), y = logFC)) +

geom_bar(stat = "identity",

aes(fill = direction),

color = "gray30",

linewidth = 0.3) +

scale_fill_manual(values = c("Up" = "#FF9999", "Down" = "#99CCFF"),

name = "Direction") +

coord_flip() +

labs(title = paste("Top 50 Genes with Largest |logFC| after", knk_gene, "Knockout"),

x = "Gene",

y = "log2(Fold Change)") +

theme_minimal() +

theme(

axis.text.y = element_text(size = 10, face = "plain"),

axis.text.x = element_text(size = 10),

axis.title = element_text(size = 12, face = "bold"),

plot.title = element_text(size = 14, face = "bold", hjust = 0.5, margin = margin(b = 10)),

legend.position = "bottom",

legend.title = element_text(face = "bold"),

panel.grid.major.y = element_blank(),

panel.grid.minor = element_blank()

) +

geom_text(aes(label = significance,

y = ifelse(logFC > 0, logFC + 0.1, logFC - 0.1)),

hjust = ifelse(all_genes$logFC > 0, 0, 1),

color = "black", size = 4)

}

\# 显示图表

print(combined_plot)

\# ==================== 保存图表为PDF ====================

\# 使用Cairo设备保存PDF

CairoPDF(

file = paste0(knk_gene, "30_significant_genes_p0.05.pdf"),

width = 14,

height = 16,

pointsize = 12

)

print(combined_plot)

dev.off()
