# Supplementary material for: TNS1/TAGLN-enriched endometrial stromal cells is associated with a persistent fibrotic microenvironment in intrauterine adhesion
Source: Front Cell Dev Biol. 2026 Aug 13;14:1810255. doi: 10.3389/fcell.2026.1810255 (PMC13518574; doi:10.3389/fcell.2026.1810255)
Supplement: Supplementary file 6 [file Supplementaryfile7.docx]

| Gene | Sequence, 5’ to 3’ |
| --- | --- |
| Human ACTA2 | Forward: CTATGAGGGCTATGCCTTGCC |
|  | Reverse: GCTCAGCAGTAGTAACGAAGGA |
| Human FN1 | Forward: AGGAAGCCGAGGTTTTAACTG |
|  | Reverse: AGGACGCTCATAAGTGTCACC |
| Human COL1A1 | Forward: GGCCCTCAAGGTTTCCAAGG |
|  | Reverse: CACCCTGTGGTCCAACAACTC |
| Human COL3A1 | Forward: TGGTCTGCAAGGAATGCCTGGA |
|  | Reverse: TCTTTCCCTGGGACACCATCAG |
| Human TNS1 | Forward: GGCCCAAAGAGCCCCATTT |
|  | Reverse: GGCCCAAAGAGCCCCATTT |
| Human TAGLN | Forward: GGCCCAAAGAGCCCCATTT |
|  | Reverse: GGCCCAAAGAGCCCCATTT |
| Human Actin | Forward: TTCCTGGGCATGGAGTCCT |
|  | Reverse: AGGAGGAGCAATGATCTTGATC |
