# Supplementary material for: TNS1/TAGLN-enriched endometrial stromal cells is associated with a persistent fibrotic microenvironment in intrauterine adhesion
Source: Front Cell Dev Biol. 2026 Aug 13;14:1810255. doi: 10.3389/fcell.2026.1810255 (PMC13518574; doi:10.3389/fcell.2026.1810255)
Supplement: Supplementary file 7 [file Supplementaryfile9.docx]

TAGLN

Tubulin

Tubulin

FN1

TNS1

TNS1

TAGLN

ACTA2

Tubulin

Tubulin

COL3A1

Tubulin

COL3A1

Tubulin

COL3A1

Tubulin

tubulin

tubulin

TNS1

TNS1

TNS1

TAGLN

tubulin
