# Supplementary material for: TNS1/TAGLN-enriched endometrial stromal cells is associated with a persistent fibrotic microenvironment in intrauterine adhesion
Source: Front Cell Dev Biol. 2026 Aug 13;14:1810255. doi: 10.3389/fcell.2026.1810255 (PMC13518574; doi:10.3389/fcell.2026.1810255)
Supplement: Supplementary file 8 [file Supplementaryfile1.docx]

12w

6w

4w

2w

Nc
