# Supplementary material for: TNS1/TAGLN-enriched endometrial stromal cells is associated with a persistent fibrotic microenvironment in intrauterine adhesion
Source: Front Cell Dev Biol. 2026 Aug 13;14:1810255. doi: 10.3389/fcell.2026.1810255 (PMC13518574; doi:10.3389/fcell.2026.1810255)
Supplement: Supplementary file 10 [file Supplementaryfile5.docx]

# HE

### Sham

### 2w

### **4w**

### 6W

### 12W **Masson NC** 2W 4w

### 6W 12W IHC_TNS1 NC 2W 4W 6W 12W IHC_TAGLN NC

### 2W 4W 6W 12W
